# miR-195-5p controls apoptotic cascade by regulating the XIAP/BCL2/Survivin network in colorectal cancer

**DOI:** 10.1038/s41420-026-03280-y

**Published:** 2026-07-28

**Authors:** Emanuele Piccinno, Viviana Scalavino, Giusy Bianco, Gianluigi Giannelli, Grazia Serino

**Affiliations:** 1 Laboratory of Molecular Medicine, National Institute of Gastroenterology S. De Bellis, IRCCS Research Hospital, Castellana Grotte, BA Italy; 2 Animal facility, National Institute of Gastroenterology S. De Bellis, IRCCS Research Hospital, Castellana Grotte, BA Italy; 3 Scientific Direction, National Institute of Gastroenterology S. De Bellis, IRCCS Research Hospital, Via Turi 27, 70013 Castellana Grotte, BA Italy

**Keywords:** Colorectal cancer, Cell death

## Abstract

Colorectal cancer (CRC) progression and therapeutic resistance are strongly associated with the ability of tumor cells to evade apoptosis through the dysregulation of key anti-apoptotic pathways. Among the molecular mechanisms involved in CRC progression, microRNAs (miRNAs) have emerged as key post-transcriptional regulators of pathways controlling tumor growth, invasion, therapeutic response, and cell survival. We previously demonstrated that miR-195-5p acts as a tumor suppressor in CRC by impairing proliferation, migration, invasion, and cell-cycle progression. However, its contribution to the regulation of apoptotic signaling remains incompletely understood. In this study, we aimed to investigate the role of miR-195-5p as a regulator of apoptotic signaling in CRC. Using membrane-based apoptosis arrays in CRC cell lines, we identified a miR-195-5p–dependent modulation of multiple apoptotic mediators, with XIAP, BCL2, and BIRC5 (Survivin) emerging as common targets across independent prediction tools. Functional validation demonstrated that miR-195-5p overexpression significantly reduced the transcript and protein levels of these anti-apoptotic factors. This molecular modulation resulted in enhanced apoptotic activity, as evidenced by increased caspase-3/7 activation, DNA fragmentation, and Annexin V-positive cell populations. Importantly, in vivo administration of miR-195-5p in an AOM/DSS mouse model confirmed the downregulation of XIAP, BCL2, and BIRC5 (Survivin) in colonic tissues, supporting its translational relevance. Overall, our findings identified miR-195-5p as a key upstream regulator of apoptosis in CRC and highlighted its potential as a promising therapeutic strategy to overcome apoptosis resistance and improve treatment outcomes.

**Figure:**
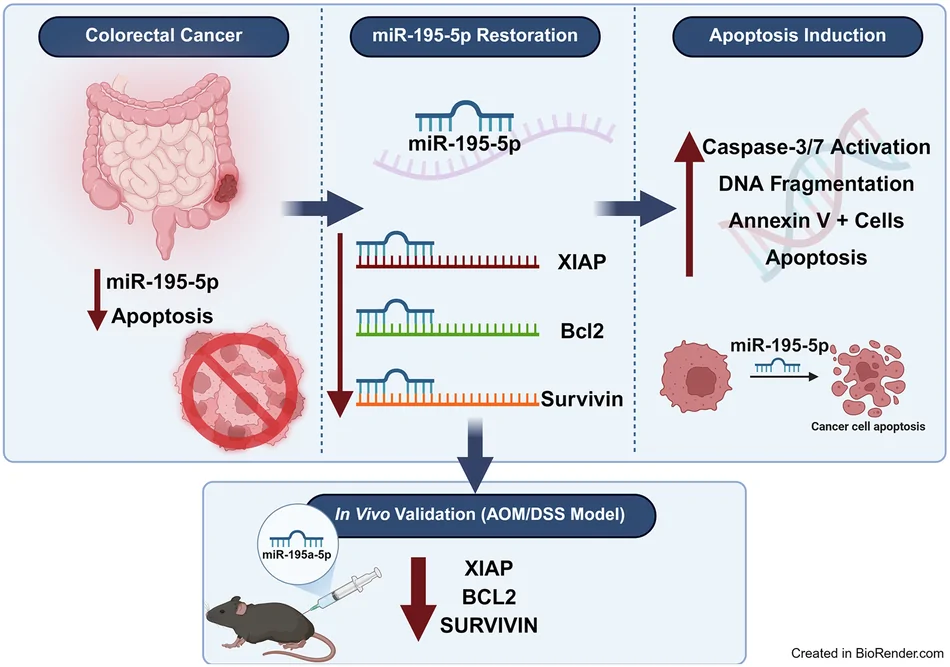

## Introduction

Colorectal cancer (CRC) remains a major clinical challenge and still represents one of the leading causes of cancer-related morbidity and mortality worldwide despite significant advances in early detection and therapeutic strategies [1]. A defining hallmark of CRC progression and treatment resistance is the ability of tumor cells to escape programmed cell death, particularly apoptosis. MicroRNAs (miRNAs) are key post-transcriptional regulators of gene expression able to modulate critical biological pathways. Altered miRNA expression is a common feature of cancer and has been implicated in wide aspects of tumor biology, including proliferation, invasion, metastasis, and apoptosis. In fact, several miRNAs have been shown to function either as oncogenes or tumor suppressors by targeting apoptosis-related genes [2,3]. Recent evidence has shown that miR-34a and miR-328 overexpression sensitizes breast cancer stem cells (BCSCs) to γ-radiation and doxorubicin by targeting BCL2 and the drug transporterABCG2, reducing viability and stemness of cancer cells as well as the drug efflux [4]. Oxaliplatin-resistant CRC cell lines display elevated XIAP expression and decreased miR-122 compared with parental cells. Restoration of miR-122 levels suppresses XIAP expression, enhances caspase-mediated signaling, and restores sensitivity of resistant CRC cells to oxaliplatin [5]. Conversely, another study reported that miR-421 was significantly upregulated in CRC tissues and cells, where it acts as an oncomiR by targeting caspase-3 (CASP3). This overexpression reduces CASP3 levels, thereby enhancing tumor cell proliferation and survival, whereas its inhibition restores CASP3 expression and promotes apoptotic cell death [6]. Similarly, miR-200 exhibited oncogenic properties promoting colon cancer aggressiveness through downregulation of PTEN and suppression of p53 activity that facilitated cell survival, invasion, and metastatic capacity [7].

Among tumor-suppressive miRNAs, miR-195-5p has emerged as a relevant regulator of CRC biology. In our previous works, we have characterized the miR-195-5p effect on CRC cells migration and invasion abilities, proliferation and viability as well as on cell cycle activity, inhibiting the transition of CRC cells from the G1 phase to the S phase [8–11]. Furthermore, we previously showed that miR-195-5p treatment markedly reduced colorectal tumor burden in an AOM/DSS murine model, supporting its therapeutic relevance in CRC [12].

Given the broad regulatory activity of miR-195-5p and the central role of apoptosis resistance in CRC, we investigated whether this miRNA also contributes to the regulation of key anti-apoptotic pathways. Apoptosis is a tightly regulated process that requires a close balance between pro-apoptotic and anti-apoptotic factors, acting through both intrinsic and extrinsic pathways [13,14]. In CRC, this balance is frequently altered with a crucial overexpression of key apoptotic inhibitors that sustain cancer cell survival. Among these molecular regulators of apoptosis, BCL2 and Inhibitor of Apoptosis Protein (IAP) family members, such as X-linked inhibitor of apoptosis protein (XIAP, also known as hILP, IAP3 and BIRC4) and BIRC5 (Survivin) play pivotal roles, preventing mitochondrial outer membrane permeabilization and affecting caspase activation. Overexpression of these factors has been consistently associated with poor prognosis, treatment resistance, and enhanced metastatic potential in CRC [15,16].

XIAP is the most potent endogenous suppressor of caspase activity and is characterized by a complex multidomain architecture composed of three baculovirus IAP repeat (BIR) domains located in the N-terminal region and a C-terminal RING finger domain that confers E3 ubiquitin ligase activity. These structural elements enable XIAP to exert a central regulatory role within the apoptotic mechanism. The BIR2 domain, together with its adjacent linker region, directly interacts and inhibits the effector caspases-3 and -7, whereas the BIR3 domain selectively binds the initiator caspase-9, preventing its activation. The RING domain further contributes to apoptosis regulation by promoting ubiquitination and proteasomal degradation of caspases and other apoptosis-related proteins [17].

XIAP as a critical checkpoint of the proteolytic cascade related to cell death is frequently overexpressed in several malignancies, including colorectal, pancreatic, lung, breast, and hematological cancers [18]. Elevated XIAP levels contribute to tumor progression by enhancing cell survival and promoting resistance to apoptosis induced by chemotherapy, radiotherapy, and targeted therapies. In several tumor types, increased XIAP expression correlates with poor prognosis, advanced disease stage, and reduced response to treatment [18–21].

Survivin, encoded by the BIRC5 gene, is one of the smallest proteins within IAP family and is composed of a single baculovirus IAP repeat (BIR) domain but lacks the C-terminal RING finger domain that is typically present in other IAP members. BIRC5 (Survivin) does not directly inhibit caspases but interferes with apoptotic signaling by forming complexes with other IAPs and regulatory proteins, thereby stabilizing anti-apoptotic signaling networks. BIRC5 (Survivin) has been reported to interact with XIAP, enhancing its stability and activity, suppressing caspase activation and promoting cell survival under stress conditions as the exposure to cytotoxic therapies [22,23]. BIRC5 (Survivin) is predominantly expressed during embryonic and fetal development, whereas its expression is minimal in most differentiated adult tissues. However, its expression becomes highly in cancer, suggesting an important role in tumor biology and a considerable interest as both prognostic biomarker and therapeutic target. Indeed, BIRC5 (Survivin) levels correlate with increased tumor aggressiveness, enhanced proliferative capacity, resistance to apoptosis, and poor clinical outcome [24,25]. BIRC5 (Survivin) also contributes to resistance to conventional therapies such as chemotherapy and radiotherapy by promoting cell survival and facilitating DNA damage tolerance [26,27].

Bcl-2 (B-cell lymphoma 2) family includes a group of 20 protein that are structurally defined by the presence of up to four Bcl-2 Homology (BH**)** domain which facilitate the complex protein-protein interactions necessary for cell death signaling. BCL-2 regulates apoptosis at the mitochondrial level by controlling mitochondrial outer membrane permeabilization (MOMP), a critical step in the intrinsic apoptotic pathway. Specifically, as a pro-survival member BCL2 prevents the release of cytochrome c and the subsequent activation of the caspase cascade [28]. Aberrant expression of BCL2 is a common feature in cancer and contributes significantly to tumor development and therapeutic resistance. Increased BCL-2 levels promote survival of malignant cells, allowing the accumulation of genetic alterations and supporting tumor progression [29]. Beyond its well-documented role in hematological malignancies like leukemias and lymphomas, elevated Bcl-2 levels are prevalent in solid tumors, including colorectal, breast, and lung cancers representing a critical barrier to the efficacy of conventional chemotherapy and ionizing radiation [30,31].

In this study, we aimed to investigate the regulatory role of miR-195-5p on apoptotic signaling in CRC. Hence, we demonstrated that miR-195-5p modulates a network of key anti-apoptotic proteins, including XIAP, BCL2, and BIRC5 (Survivin). We further assessed the impact of miR-195-5p on programmed cell death in vitro and validated the clinical relevance of our findings in an established AOM/DSS-induced murine model of colorectal carcinogenesis. This study provides new insights into the molecular regulation of apoptosis and identifies potential therapeutic targets for overcoming apoptosis resistance in CRC.

## Results

### miR-195-5p modulates apoptotic signaling networks in CRC cells

In order to investigate the effect of miR-195-5p on the apoptotic signaling as well as to identify molecular drivers of apoptosis resistance in CRC, we assessed a membrane-based protein array that allowed a simultaneous overview of the key apoptotic mediators (Fig. 1A).

**Fig. 1:**
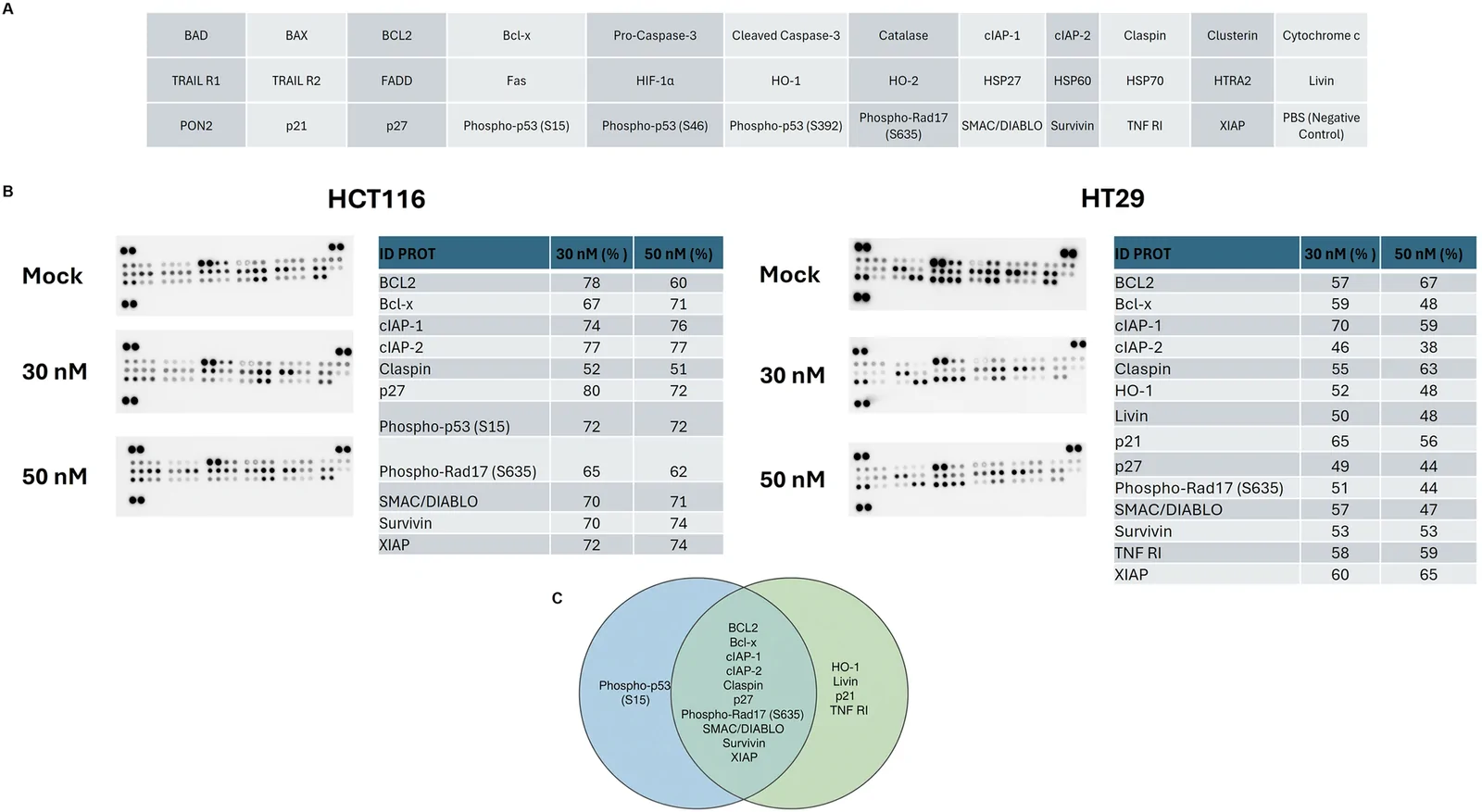
miR-195-5p modulates multiple apoptotic mediators in CRC cells. **A** List of the 35 apoptotic proteins included in the membrane-based human apoptosis array. **B** Representative dot-blot images and quantitative analysis of apoptotic protein expression in HCT116 (left) and HT29 (right) cells transfected with 30 nM or 50 nM miR-195-5p mimic, compared with mock-treated controls. For each cell line, the corresponding tables report proteins significantly modulated upon miR-195-5p overexpression, expressed as percentage change relative to the mean of mock. **C** Venn diagram illustrates the overlap of significantly regulated proteins between HCT116 and HT29 transfected with miR-195-5p mimic. A shared apoptotic signature was observed, including BCL2, Bcl-x, cIAP-1, cIAP-2, Claspin, p27, phospho-Rad17 (S635), SMAC/DIABLO, Survivin, and XIAP.

Our analysis revealed that miR-195-5p mimic transfection at 30 nM and 50 nM concentrations induced a distinct modulation of apoptosis-related proteins in both HT29 and HCT116 cell lines. Among the 35 membrane-associated apoptotic mediators examined, 11 proteins showed statistically significant changes in HCT116 cells and 14 in HT29 cells compared with control conditions (Fig. 1B). Notably, a substantial overlap of a core set of proteins between the two cell lines was observed including BCL2, Bcl-x, cIAP-1, cIAP-2, Claspin, p27, phospho-Rad17 (S635), SMAC/DIABLO, Survivin, and XIAP. In contrast, phospho-p53 (S15) was exclusively modulated in HCT116 cells, whereas HO-1, Livin, p21, and TNF RI were uniquely altered in HT29 cells. Overall, these findings suggest that miR-195-5p affects apoptosis, indicating a cell line-specific effect but also a shared miR-195-5p–dependent apoptotic signature across the two CRC models.

### miR-195-5p is a potential regulator of XIAP, BCL2 and BIRC5 (Survivin)

To further explore the biological relevance of the apoptotic mediators modulated by miR-195-5p, we performed target prediction analyses using established miRNA target prediction tools, including miRabel, miRWalk, Tarbase, miRGator, miRmap and TargetScan (Fig. 2A). Among the subset of common apoptotic mediators identified in both cell lines from the membrane-based apoptosis array, XIAP, BCL2, and BIRC5 (Survivin) emerged as putative targets of miR-195-5p across at least 3 databases (Fig. 2B). Since we previously proved that miR-195-5p was strongly downregulated in CRC [12], we hypothesized that miR‑195‑5p loss may contribute to the dysregulation of key apoptotic mediators that are typically upregulated in CRC.

**Fig. 2:**
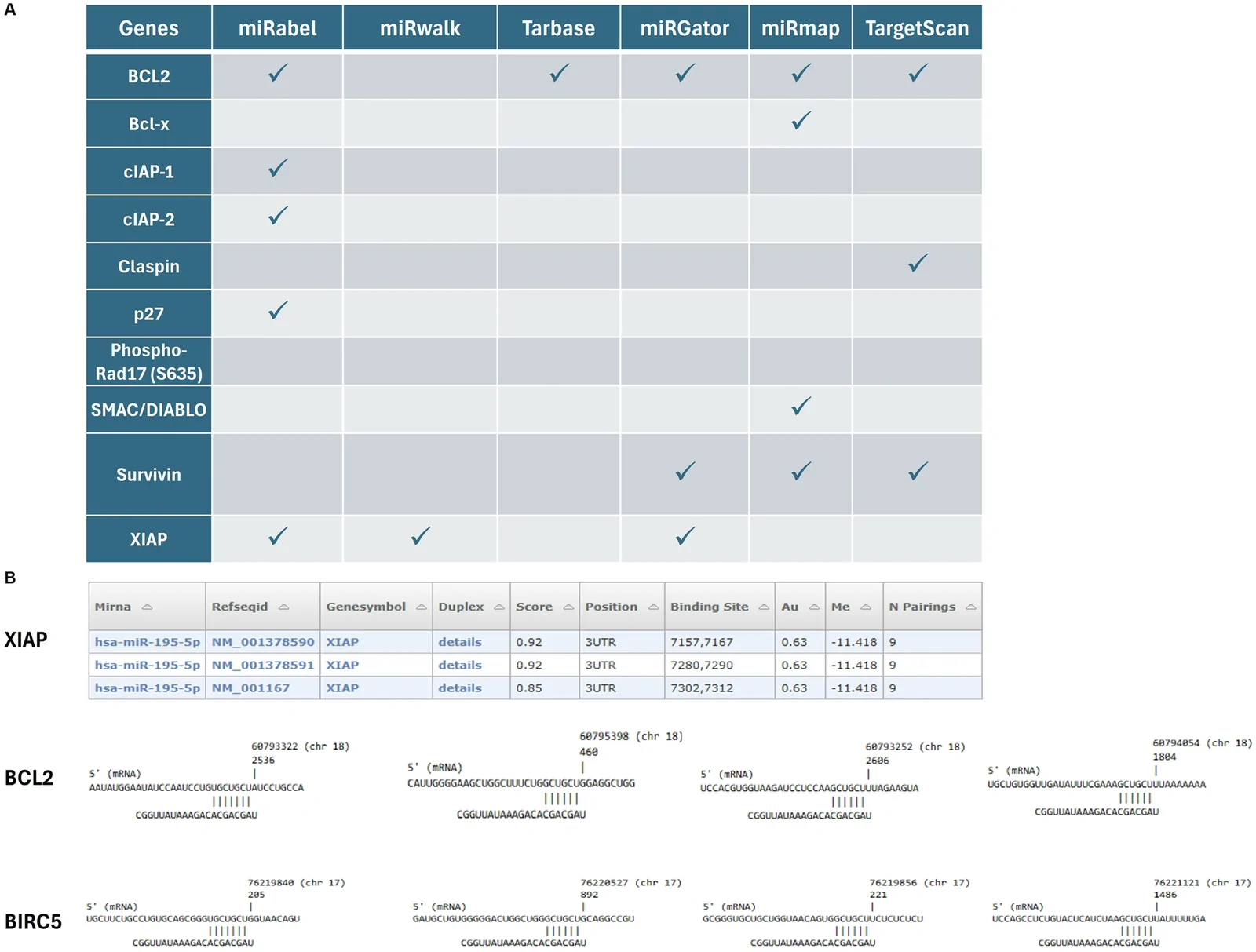
miR-195 putative target prediction. **A** Workflow of the target prediction analysis using established tools, including miRabel [44], miRWalk [45], Tarbase [46], miRGator [47], miRmap [48], and TargetScan [49]. **B** Identification of XIAP, BCL2, and BIRC5 (Survivin) as putative targets of miR-195-5p across multiple databases among the common apoptotic mediators identified in the protein array. Notably, the analysis revealed conserved miR-195-5p recognition elements within the 3′ untranslated regions (3′UTRs) of these genes that were selected for further experimental validation.

### miR-195-5p downregulates XIAP, BCL2, and BIRC5 (Survivin) expression in vitro

Based on the bioinformatic predictions, we carried out in vitro experiments to verify the miR-195-5p effectiveness on the regulation of the identified apoptotic mediators. Hence, HT29 and HCT116 cells were transiently transfected with miR-195-5p mimic and the expression of XIAP, BCL2, and BIRC5 (Survivin) was assessed at both transcriptional and protein levels. Quantitative RT‑PCR revealed a marked reduction of all three transcripts following miR‑195‑5p overexpression in both cell lines, indicating that miR‑195‑5p effectively suppresses the expression of these genes also at the mRNA level (Fig. 3).

**Fig. 3:**
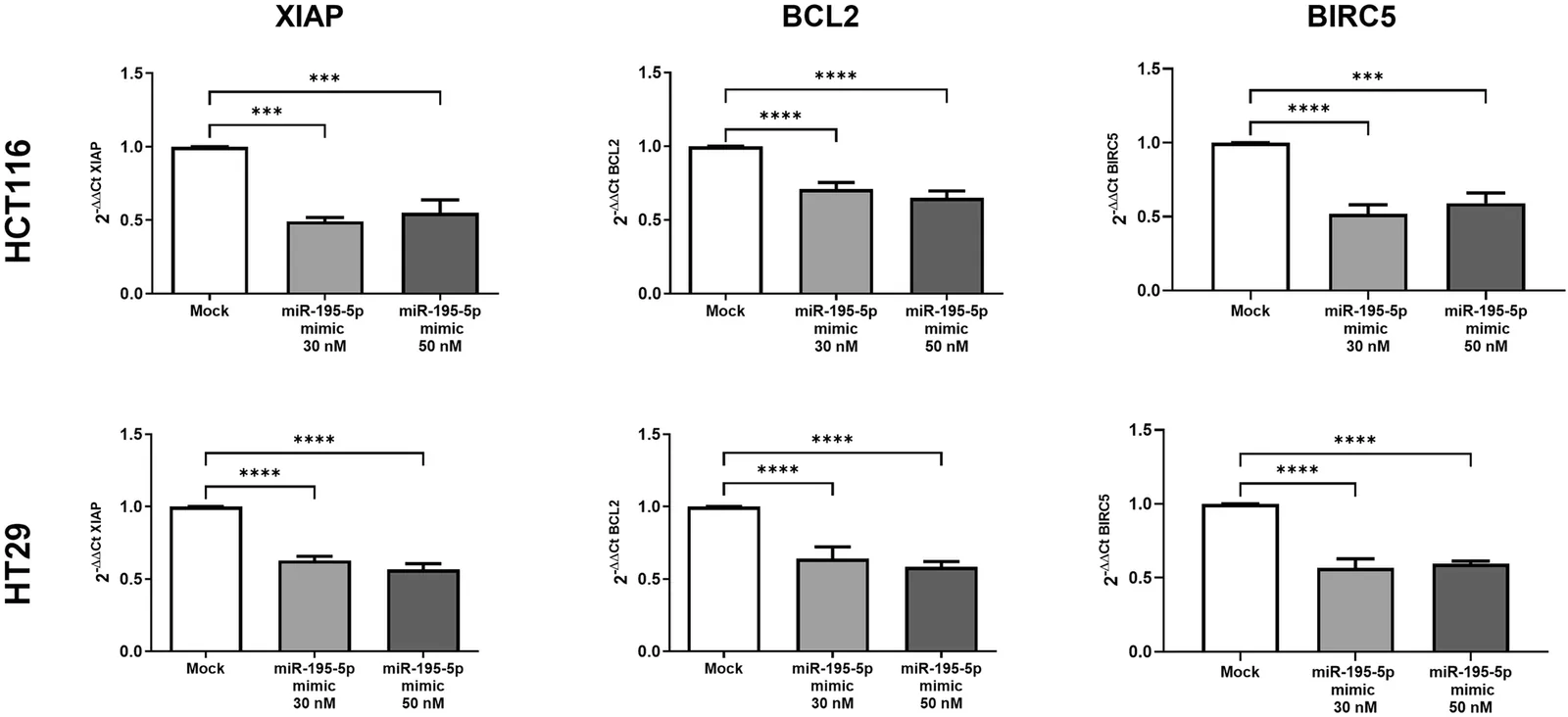
miR-195-5p reduces XIAP, BCL2, and BIRC5 (Survivin) transcript levels in CRC cells. Quantitative RT-PCR analysis showing a significant reduction in XIAP, BCL2, and BIRC5 (Survivin) mRNA levels in HT29 and HCT116 cells transfected with miR-195-5p mimic compared with mock controls. Relative mRNA expression was calculated using the 2^–ΔΔCt^ method, normalized to GAPDH and expressed as fold change relative to mock controls. Data represent mean ± SEM of at least three independent experiments. ***p < 0.001, ****p < 0.0001.

Consistent with these findings, Western blot analyses demonstrated a significant decrease of XIAP, BCL2, and BIRC5 (Survivin), extending the miR‑195‑5p regulatory effect on protein levels. Indeed, the induction of miR-195-5p at 30 nM and 50 nM concentrations led to a remarkable reduction of all three effectors in both HT29 and HCT116 cells (Fig. 4).

**Fig. 4:**
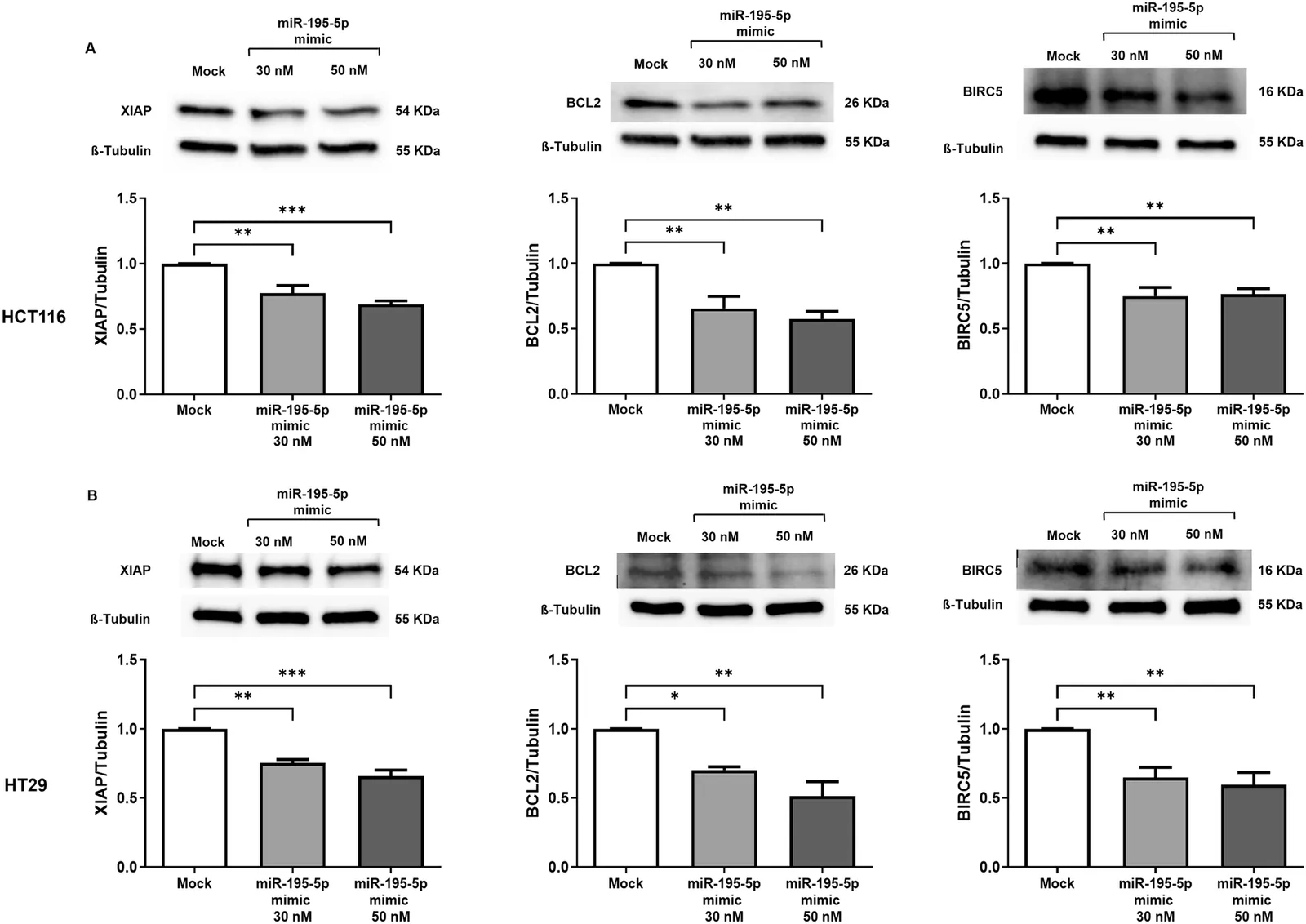
miR-195-5p reduces the protein expression of XIAP, BCL2, and BIRC5 (Survivin) in CRC cell lines. Representative Western blot images and densitometric quantification of XIAP, BCL2, and BIRC5 (Survivin) protein levels in HCT116 (**A**) and HT29 (**B**) cells 48 h after transfection with miR-195-5p mimics (30 nM and 50 nM). miR-195-5p overexpression markedly reduced all three anti-apoptotic proteins. β-Tubulin was used as a loading control to normalize protein levels. Data are presented as mean ± SEM of independent replicates. *p < 0.05, **p < 0.01, ***p < 0.001.

Collectively, these results highlighted that miR-195-5p functionally modulated the expression of key anti-apoptotic mediators in CRC cells.

### miR-195-5p overexpression enhances apoptotic activity in CRC cells

To determine whether the repression of XIAP, BCL2, and BIRC5 (Survivin) upon miR-195-5p overexpression trigger a functional apoptotic response, we performed a series of complementary assays and examined different stages of the apoptotic cascade. Caspase-3/7 activation is widely considered as the most important molecular marker for apoptotic cell death [32]. Since Bcl-2 prevents the upstream initiation of the apoptotic cascade, while XIAP and BIRC5 (Survivin) act as direct inhibitors of executioner caspases, we assessed caspase‑3/7 activity to determine whether their miR-195-5p-mediated downregulation effectively triggered a functional apoptotic response.

Assessment of caspase-3/7 enzymatic activity revealed a significant increase in caspase activation in both HT29 and HCT116 cells following miR-195-5p mimic transfection, indicating the activation of the apoptotic effector machinery. The rise in caspase-3/7 activity was observed across different mimic concentrations and correlated with the downregulation of anti-apoptotic components that act as upstream negative regulators of effector caspases (Fig. 5).

**Fig. 5:**
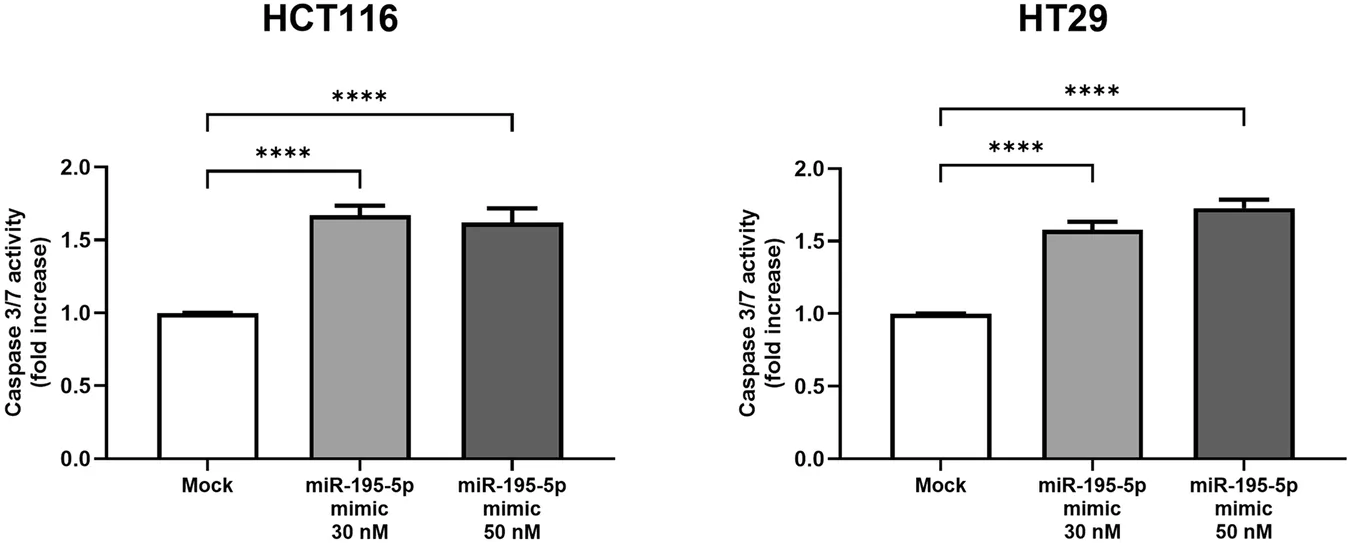
miR-195-5p overexpression triggers Caspase-3/7 activation. Assessment of Caspase-3/7 enzymatic activity using a luminescent assay upon miR-195-5p overexpression Both transfected cell lines displayed a significant increase in caspase-3/7 activation compared to mock control. Luminescence values are expressed as fold increase relative to mock-treated control cells. Data are shown as mean ± SEM. ****p < 0.0001.

To further validate these findings, we evaluated DNA fragmentation through TUNEL staining. Consistent with the caspase 3/7 activity, in both CRC cell lines, the gain of miR-195-5p at 30n nM and 50 nM concentrations induced a substantial, dose-dependent increase in TUNEL-positive cells, confirming enhanced apoptotic DNA damage (Fig. 6).

**Fig. 6:**
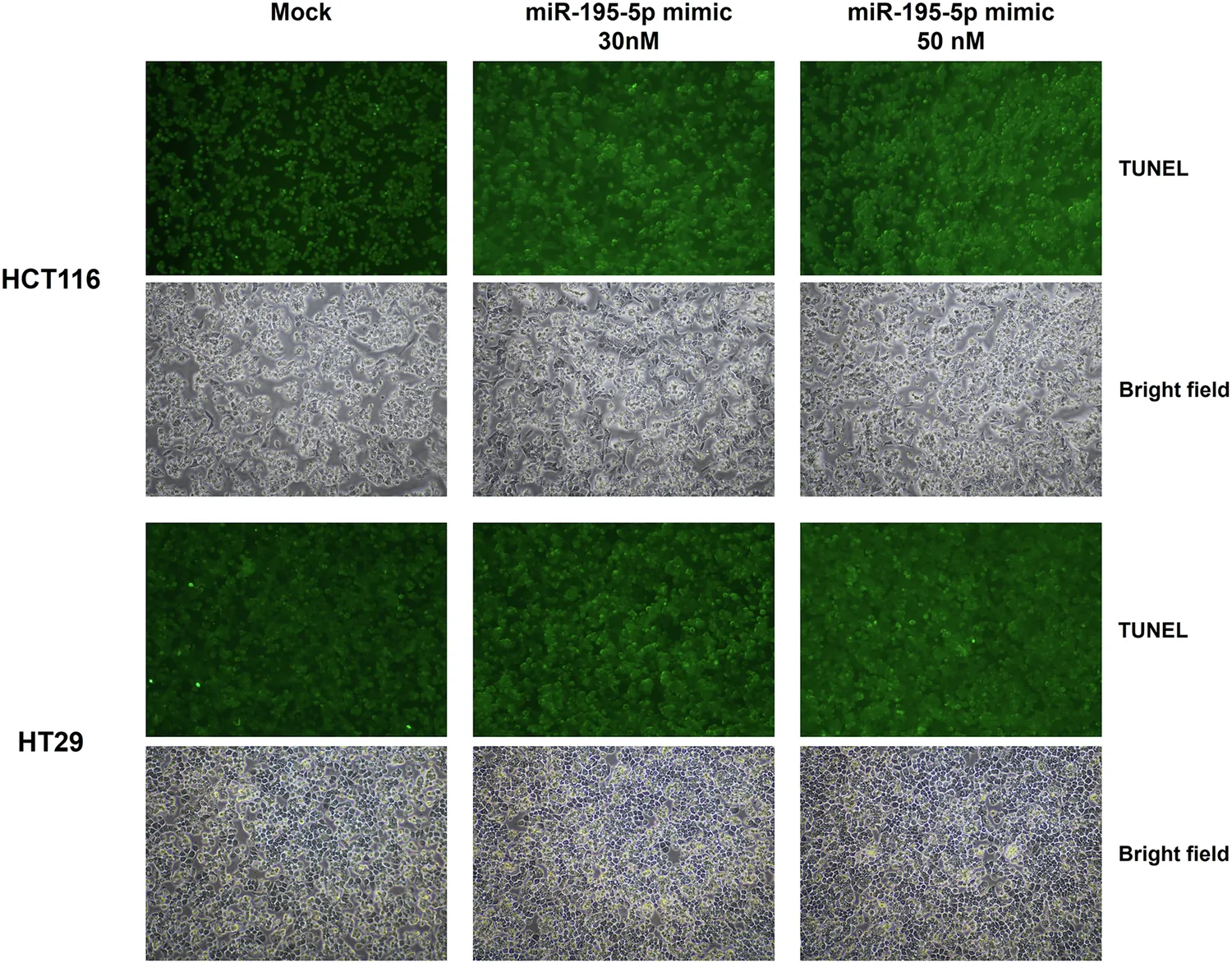
miR-195-5p induces DNA fragmentation in CRC cells. Representative fluorescence microscopy images and quantification of TUNEL-positive nuclei in HCT116 and HT29 cell lines following miR-195-5p mimic transfection (30 nM and 50 nM). TUNEL staining revealed a higher proportion of apoptotic nuclei in miR-195-5p-overexpressing cells relative to controls demonstrating enhanced apoptotic DNA damage following miRNA mimic transfection in a dose-dependent manner.

Similarly, Annexin V staining was then performed to quantify the distribution of live, early apoptotic, and late apoptotic populations. After mimic transfection, both HCT116 and HT29 cells showed a significant redistribution of viability profiles compared to mock controls (*p* < 0.05). Notably, increasing the intracellular miR‑195‑5p levels (30 nM and 50 nM) resulted in a marked shift toward apoptosis, with a clear reduction in the proportion of viable cells and a concomitant raise of the apoptotic events in both early and late apoptotic fractions (Fig. 7).

**Fig. 7:**
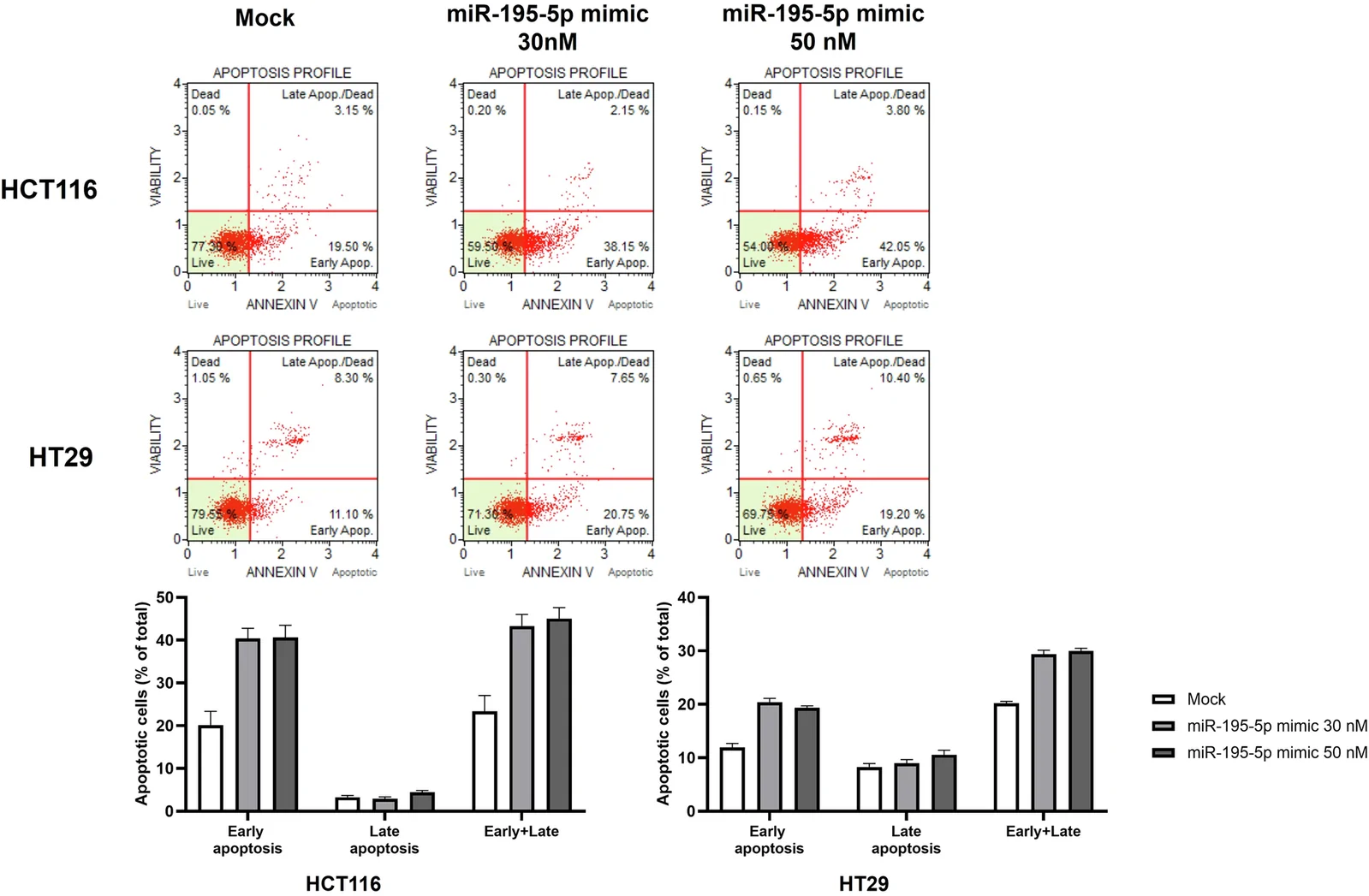
miR-195-5p promotes apoptosis in CRC cells. Representative Annexin V flow cytometry plots and quantification of viable, early apoptotic, and late apoptotic cells. miR-195-5p mimic transfection significantly increased both early and late apoptosis, with a marked reduction in viable populations in both HCT116 and HT29 cell lines. Data were normalized on mock control (*n* = 3; mean ± SEM).

All together, these findings confirmed that miR-195-5p enhances apoptotic susceptibility in CRC cells.

### miR-195-5p suppresses XIAP, BCL2 and BIRC5 (Survivin) in vivo

To assess the translational relevance of our in vitro findings, we examined the expression of XIAP, BCL2, and BIRC5 (Survivin) in colon tissues from a previously established AOM/DSS mouse model. As described in our publication, mice (*n* = 28) were divided into a control group (vehicle; *n* = 14) and a treatment group receiving intraperitoneal administration of miR-195-5p mimic (*n* = 14) twice a week until sacrifice [12]. Gene-expression analysis performed on colon segments revealed a marked downregulation of *Xiap, Bcl2*, and *BIRC5 (Survivin)* in miR-195-5p treated mice compared with vehicle controls, indicating that miR-195-5p delivery effectively suppresses these anti-apoptotic mediators in vivo (Fig. 8).

**Fig. 8:**
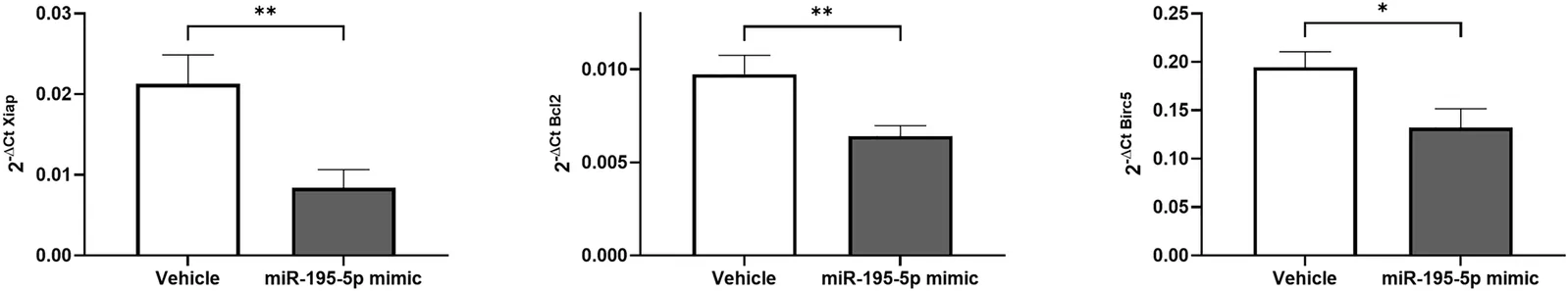
In vivo suppression of XIAP, BCL2, and BIRC5 (Survivin) mRNA in AOM/DSS-treated mice. Gene expression analysis of *Xiap, Bcl2, and Birc5* (Survivin) in colon tissues from AOM/DSS-treated mice receiving miR-195-5p mimic or vehicle. miR-195-5p administration significantly reduced the expression of all three anti-apoptotic mediators, demonstrating effective in vivo modulation. Expression data were normalized on housekeeping gene *Gapdh* and are shown as mean ± SEM. * *p* < 0.01, ** *p* < 0.001.

In accordance with transcriptional data, immunofluorescence staining of colon tissue showed a pronounced decrease of XIAP, BCL2, and BIRC5 (Survivin) in miR-195-5p–treated group (Fig. 9). In vehicle-treated animals, all three proteins displayed strong and widespread fluorescence signals across the epithelial section, indicative of high basal expression of these anti-apoptotic mediators. In contrast, colon sections from mice exposed to miR-195-5p mimic showed a marked attenuation of staining. This reduction was consistently observed across all three markers, suggesting that miR-195-5p in vivo administration effectively suppresses these anti-apoptotic proteins.

**Fig. 9:**
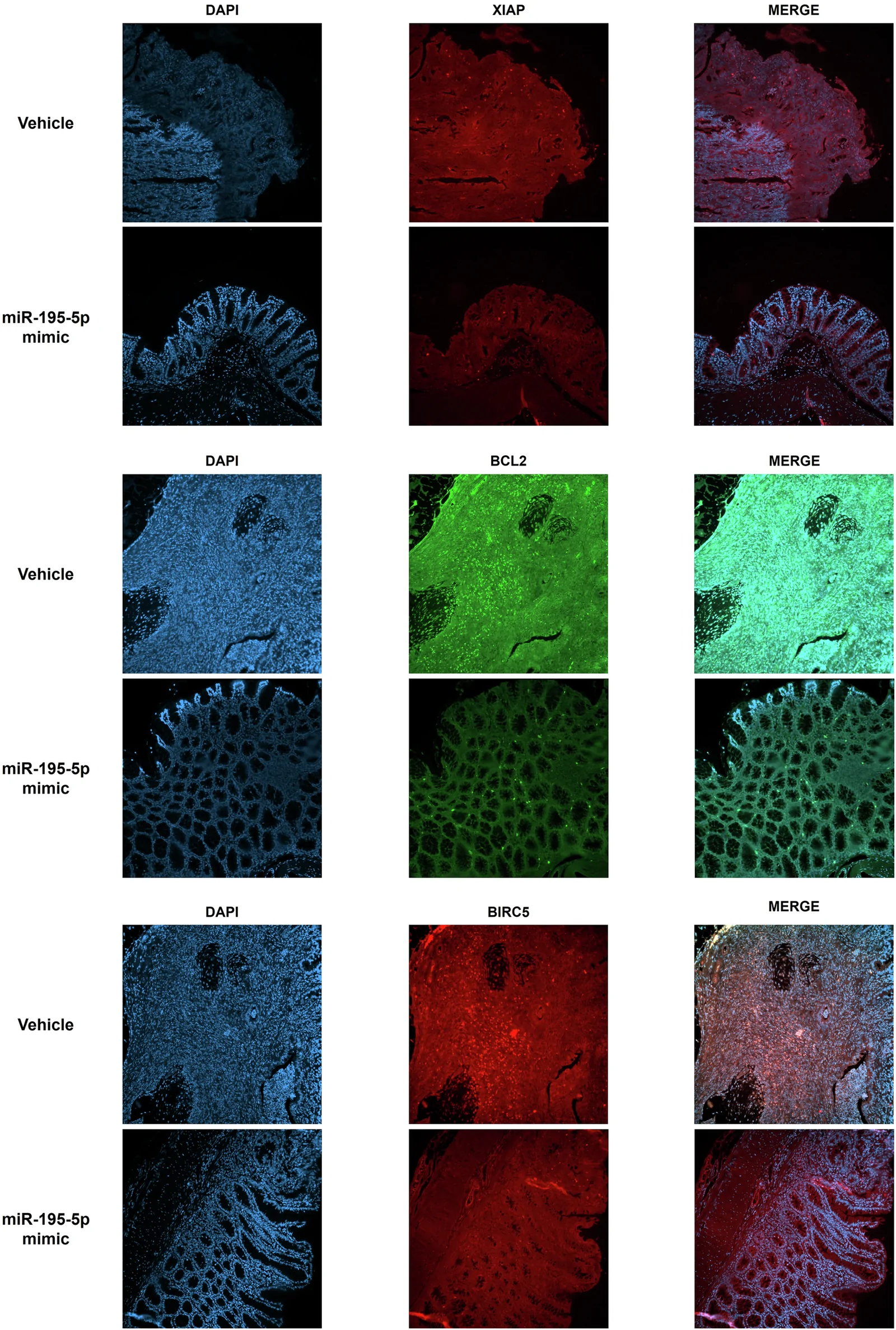
miR-195-5p reduces anti-apoptotic proteins expression in CRC tissues. Representative immunofluorescence staining of XIAP, BCL2, and BIRC5 (Survivin) (green/red) in colon sections from vehicle-treated and miR-195-5p-treated AOM/DSS mice. Nuclei were counterstained with DAPI (blue). A pronounced decrease in fluorescence intensity for all three markers was observed in the miR-195-5p-treated group relative to the strong basal expression in vehicle controls. Images were acquired at 20× magnification.

Collectively, these findings reinforce the biological significance of XIAP, BCL2, and BIRC5 (Survivin) as critical survival mediators in CRC and support the potential relevance of miR-195-5p in modulating apoptosis pathway.

## Discussion

A key driver of CRC initiation and progression is the evasion of apoptosis by malignant cells, a process that facilitates tumor development and compromises therapeutic efficacy. Both the intrinsic (mitochondrial) and extrinsic (death receptor-mediated) apoptotic pathways are frequently impaired in CRC, leading to reduced sensitivity to cell death signals. This impairment is often driven by genetic and epigenetic alterations affecting critical regulators of apoptosis, including members of the IAP and BCL-2 family, caspases, and death receptors [14]. Among the main molecular regulators involved, XIAP, BCL2 and BIRC5 (Survivin) play critical roles in suppressing caspase activation and preventing mitochondrial outer membrane permeabilization. The aberrant activity of these components is often associated with impaired apoptotic responses and tumor growth. For instance, the suppression of XIAP and NF-κB signaling reduced pro-survival trigger and enhanced apoptotic susceptibility in 5-fluorouracil-resistant CRC cells [33]. A recent work highlighted that HNPMI, a novel EGFR inhibitor exhibited potential anti-cytotoxicity and anti-proliferative activity in CRC cells by inducing the intrinsic apoptotic pathway and oncogenesis pathway through the regulation of BCL-2 and p53 signaling pathways. Another study has identified CCT6A as an additional regulator of apoptotic resistance in colon cancer, acting through modulation of BIRC5 (Survivin). CCT6A promotes tumor cell proliferation by regulating BIRC5 expression in a p53-dependent manner, and its inhibition leads to reduced BIRC5 levels and impaired cancer cell growth [34].

Emerging evidence highlights that post-transcriptional regulators such as miRNAs impact apoptosis evasion by targeting components of these pathways, further affecting CRC cell survival and disease evolution [35]. In fact, miRNA research has become a cornerstone for the next generation of molecular therapies for their abilities in post transcriptional regulation of gene expression that allows to control several biological effectors. miR -766 was identified as a critical tumor suppressor in colon cancer and was significantly downregulated in affected patients. It was found that overexpressing miR-766 inhibits cell migration and viability increasing caspase-3/9 activity and triggers apoptosis by suppressing the MDM4/p53 signaling pathway. Consequently, higher levels of this miRNA are strongly correlated with improved overall and disease-free survival rates [36]. Instead, miR-21 was significantly upregulated in CRC tissues and correlates with advanced disease stages and poor prognosis. Through functional assays assessed on CRC cell lines, the authors observed that the overexpression of miR-21 inhibited apoptosis and promoted cell proliferation, invasion, and the growth of xenograft tumors in mice via PTEN suppression [37]. Shang and co-workers observed that 5-FU treatment led to a miR-23a upregulation which in turn suppresses the Apoptotic Protease-Activating Factor-1 (APAF-1), a protein essential for the mitochondrial death pathway. They have discovered that the miR-23a inhibition can restore the APAF-1/caspase-9 signaling axis, effectively re-sensitizing resistant cells to 5-FU-induced programmed cell death [38]. These observations underscore how the dysregulation of specific miRNA-mRNA axes drives apoptosis resistance in CRC.

In our previous papers, we highlighted a pronounced downregulation of miR-195-5p in CRC patients and showed that the raise of its expression critically regulated key oncogenic drivers suppressing migration/invasion, proliferation as well as inducing cell cycle arrest in CRC cell lines [8–12]. Furthermore, the miR-195-5p in vivo treatment markedly reduced the number of colorectal tumors and impaired their progression in AOM/DSS-treated mice [12]. Importantly, the tumor-suppressive role of miR-195-5p in CRC is not limited to apoptosis regulation. Previous studies, including our works, have identified several biologically relevant targets that contribute to CRC progression. Among these, GDPD5 has been validated as a direct miR-195-5p target and has been associated with enhanced metastatic potential, stemness features, and chemoresistance in colorectal cancer. The ability of miR-195-5p to suppress GDPD5 expression further supports its role as a master regulator of aggressive tumor phenotypes [39].

In addition, accumulating evidence indicates that miR-195-5p regulates pathways linked to epithelial organization and Wnt/β-catenin-associated signaling. We previously demonstrated that miR-195-5p targets γ-catenin (JUP), a key component of desmosomal junctions and a functional modulator of β-catenin transcriptional activity [8,12]. Moreover, we identified additional miR-195-5p targets including PNN, KRT23 and KRT80, whose deregulation contributes to CRC cell proliferation, migration, invasion and cell-cycle progression [9–11]. Together, these findings indicate that miR-195-5p controls multiple interconnected oncogenic networks involved in cell adhesion, epithelial plasticity, tumor growth and metastatic dissemination.

In this study, our findings reinforce the concept that dysregulation of apoptotic pathways plays a central role in CRC pathogenesis, particularly through the alteration of key apoptotic regulators. Indeed, our results derived from the protein apoptosis array analysis underscored that miR-195-5p overexpression produces a coordinated modulation of multiple apoptotic mediators in CRC cells, indicating that this miRNA acts as a broad upstream regulator of apoptosis. Beyond XIAP, BCL2 and BIRC5 (Survivin), the apoptosis array revealed the modulation of several additional proteins, including Bcl-x, cIAP-1, cIAP-2, Claspin, p27, phospho-Rad17 (S635), SMAC/DIABLO, HO-1, Livin, p21 and TNF-RI. Although these molecules were not further validated in the present study, their deregulation provides additional insight into the broader biological effects exerted by miR-195-5p. Notably, many of these factors are involved in the regulation of cell survival, DNA damage response, cell-cycle control and stress-adaptation pathways, suggesting that miR-195-5p may influence multiple interconnected processes beyond the canonical apoptotic cascade. For example, cIAP-1, cIAP-2 and Livin belong to the inhibitor of apoptosis protein (IAP) family and cooperate with XIAP in the suppression of caspase activation [40], whereas Claspin and phospho-Rad17 are involved in DNA damage checkpoint signaling [41]. Similarly, p21 and p27 are key regulators of cell-cycle progression, consistent with our previous observations demonstrating a role for miR-195-5p in cell-cycle arrest [11]. These findings support the hypothesis that miR-195-5p functions as a pleiotropic tumor suppressor capable of coordinately regulating multiple pathways involved in CRC progression.

Consistent with these results, target prediction analyses identified XIAP, BCL2, and BIRC5 (Survivin) as common putative targets of miR-195-5p across multiple independent databases, further supporting the involvement of this miRNA in the regulation of apoptotic pathways and reinforcing the mechanistic insights suggested by our proteomic array. Importantly, the experimental validation confirmed that miR-195-5p overexpression reduced both transcript and protein levels of XIAP, BCL2, and BIRC5 (Survivin) in CRC cells, highlighting a molecular mechanism that was able to affect multiple apoptotic pathways simultaneously and to potential overcome the survival strategies typical of malignancies. In addition, to functionally characterize the impact of miR-195-5p on apoptosis induction we employed a panel of complementary assays exploring key phases of the cell death process. Indeed, the downregulation of XIAP, BCL2, and BIRC5 (Survivin) induced by the gain of miR-195-5p also lead to a clear activation of the apoptotic machinery, as shown by increased caspase-3/7 activity, enhanced DNA fragmentation, and a significant enrichment of Annexin V-positive cell populations that underscored a consistent shift from viable to early and late apoptotic cells. These complementary assays demonstrated that miR-195-5p restoration not only modulate apoptotic marker expression but actively promoted a coordinated execution of the cell death program thereby restoring cell-death susceptibility in CRC cells. Finally, the in vivo validation performed in AOM/DSS-treated mice supported the translational relevance of our data. The marked reduction of XIAP, BCL2, and BIRC5 (Survivin) transcripts in colonic tissues from miR-195-5p-treated mice demonstrated the miR-195-5p effectiveness in the suppression of key survival factors within the native tumor microenvironment. The reduction of protein expression observed by immunofluorescence supported the idea that miR‑195‑5p administration achieved effective delivery to the target tissue and exerted a biological impact on apoptotic regulation. Notably, while our primary mechanistic focus centered on classical apoptosis, many of the identified targets, including XIAP, cIAP1/2, TNF-RI, BCL-xL, and HO-1, are increasingly recognized as crucial hubs bridging apoptosis with alternative regulated cell death modalities and broader cellular stress-response networks [42]. Recent studies have highlighted the relevance of ferroptosis and necroptosis in CRC progression and therapeutic response, suggesting that modulation of these pathways may influence tumor susceptibility to treatment. In particular, the modulation of TNF-RI and cellular inhibitors of apoptosis (XIAP and cIAP1/2) represents a critical molecular axis capable of shifting from survival or apoptosis to necroptosis [43]. Under conditions where IAPs are depleted or suppressed, TNF-RI signaling can actively promote the assembly of the necrosome complex involving RIPK1, RIPK3, and MLKL, which are key executors of necroptotic cell death in CRC biology [43]. Similarly, BCL-xL and Heme Oxygenase-1 (HO-1) play pivotal, yet complex, roles in regulating oxidative stress and iron homeostasis. HO-1, for instance, serves as a dual modulator of ferroptosis; its regulation directly impacts the intracellular labile iron pool, lipid peroxidation, and reactive oxygen species (ROS) accumulation [43]. Although the present study was not designed to investigate these mechanisms and our functional analyses were focused on apoptotic endpoints, the broad spectrum of proteins affected by miR-195-5p raises the possibility that its biological activity may extend beyond apoptosis regulation alone. Future studies should therefore determine whether restoration of miR-195-5p expression influences ferroptosis or necroptosis in CRC and whether these mechanisms contribute to the overall tumor-suppressive activity of this miRNA.

Collectively, our findings identified miR-195-5p as a key upstream regulator of apoptosis in CRC, capable of modulating multiple anti-apoptotic pathways via XIAP, BCL2, and BIRC5 (Survivin) modulation. These results not only provide mechanistic insight into the pro-apoptotic role of miR-195-5p but also highlight its potential as a promising therapeutic candidate to restore apoptotic sensitivity and improve treatment response in CRC. Future investigations should focus on validating the therapeutic potential of miR-195-5p in preclinical models as well as on optimizing delivery strategies. Moreover, evaluating miR-195-5p-based therapy in combination with standard chemotherapeutic may provide new opportunities to overcome drug resistance and inhibit tumor growth.

In conclusion, our study provided evidence that miR-195-5p acts as a potent tumor suppressor in CRC by restoring apoptotic sensitivity. Through the coordinated downregulation of the XIAP/BCL2/BIRC5 (Survivin) axis, miR-195-5p triggered the caspase-dependent death cascade and reduced tumor cell viability activating the apoptotic cascade. We demonstrated that these anti-apoptotic mediators are key drivers of CRC survival and that miR-195-5p effectively reverses their overexpression in both CRC cell lines and AOM/DSS-treated mice. These findings establish miR‑195‑5p as a promising molecular tool to overcome apoptosis resistance and improve therapeutic sensitivity in CRC, supporting its future exploration in translational and therapeutic applications.

## Materials and methods

### Cell culture

Human CRC cell lines HCT116 and HT29 were obtained from the American Type Culture Collection (ATCC, Manassas, VA, USA). Both cell lines were maintained in Dulbecco’s Modified Eagle Medium (DMEM) supplemented with 10% heat‑inactivated fetal bovine serum (FBS), 1% penicillin–streptomycin, 10 mM HEPES, and 1 mM sodium pyruvate (all reagents from Thermo Fisher Scientific, Waltham, MA, USA). Cultures were kept in a humidified incubator at 37 °C with 5% CO₂ and routinely passaged to maintain exponential growth.

For functional experiments, cells were seeded at appropriate densities in 6‑ or 12‑well plates and transfected at confluence with miR‑195‑5p mimic (Life Technologies, Carlsbad, CA, USA) at 30 nM or 50 nM, using TKO transfection reagent (Mirus Bio LLC, Madison, WI, USA), following the manufacturer’s instructions. In all transfection experiments, a mock control, representing the only transfection reagent, was included.

### RNA extraction and quantitative real‑time PCR

Total RNA was extracted from cells and mouse colon tissues using TRIzol reagent (Invitrogen, Carlsbad, CA, USA Thermo Fisher Scientific, Waltham, MA, USA). Cells were plated in 12-well plates and then lysed for RNA extraction 24 h post transfection. RNA concentration and purity were determined using a NanoDrop ND‑2000 spectrophotometer (Thermo Fisher Scientific, Waltham, MA, USA).

For cDNA synthesis, iScript Reverse Transcription Supermix (Bio‑Rad Laboratories, Hercules, CA, USA) was used according to the manufacturer’s instructions. Quantitative PCR was carried out using SsoAdvanced Universal SYBR Green Supermix (Bio‑Rad Laboratories, Hercules, CA, USA) on a CFX96 Real‑Time PCR System (Bio‑Rad Laboratories, Hercules, CA, USA).

Gene‑specific human or mice primers for XIAP, BCL2, BIRC5 (Bio‑Rad Laboratories, Hercules, CA, USA) and GAPDH (Qiagen, Hilden, Germany) asreference gene were also used. All experiments were performed in at least three independent biological replicates and the relative mRNA expression levels were calculated using the 2^^–ΔΔCt^ method.

### Protein extraction, apoptotic protein profiling and western blotting

Total protein was isolated using T‑PER Extraction Reagent (Thermo Fisher Scientific, Waltham, MA, USA) supplemented with a protease inhibitor cocktail (Sigma‑Aldrich, St. Louis, MO, USA) and then the protein concentration was assessed by Bradford assay (Bio‑Rad Laboratories, Hercules, CA, USA).

To evaluate the expression of multiple apoptotic markers simultaneously, a membrane-based human apoptosis antibody array (including 35 specific mediators) was utilized (R&D Systems, Minneapolis, MN, USA). Briefly, protein extracts from HCT116 and HT29 cells transfected with miR-195-5p mimics or mock controls were incubated with the array membranes. Detection was performed via chemiluminescence, and spot intensities were quantified using densitometric analysis by means of Image Lab Software version 6.1 (Biorad Laboratories, Hercules, CA, USA). Data were expressed as a percentage relative to the mean of mock group.

For western blot analysis, an equal amount of proteins were denatured at 100 °C for 5 min in Laemmli buffer and separated using 4–20% TGX Stain‑Free SDS‑PAGE gels (Bio‑Rad Laboratories, Hercules, CA, USA). Proteins were transferred onto PVDF membranes (0.2 µm pore size) by means of Trans-Blot Transfer Turbo (Bio‑Rad Laboratories, Hercules, CA, USA) according to manufactures instructions. Primary antibodies against XIAP (A15403, Antibodies, Cambridge, United Kingdom), BCL2 (15071, Cell Signaling, Technology, Danvers, MA, USA), Survivin (A2808, Cell Signaling, Technology, Danvers, MA, USA), and β‑Tubulin (loading control, sc-166729, Santa Cruz Biotechnology, Inc., Heidelberg, Germany) as well as secondary antibodies of Goat Anti- mouse IgG-(H + L)-HRP conjugate (170-6516, Biorad Laboratories, CA, USA) and Goat Anti-rabbit IgG-(H + L)-HRP conjugate (#31466, Invitrogen, Carlsbad, CA, USA) were incubated using an iBind Flex Western Device (Thermo Fisher Scientific, Waltham, MA, USA). Detection was assessed with ChemiDoc Imaging System (Bio‑Rad Laboratories, Hercules, CA, USA) and the blot intensities were quantified using ImageJ v1.54 d.

### Caspase‑3/7 activity assay

Caspase activity was measured using a luminescent assay according to the manufacturer’s protocol (e.g., Caspase‑Glo® 3/7 Assay System, Promega, Madison, WI, USA). Briefly, cells were plated in 6-well plates and transfected with miR‑195‑5p mimic at 30 nM and 50 nM concentrations. After 48 h transfection cells were detached using 0.05% trypsin, counted by means of the Countess™ 3 Automated Cell Counter (Thermo Fisher Scientific, Waltham, MA, USA), and re-plated in 96-well plates in a final volume of 100 μl for 24 h before reagent addition. Luminescence was recorded using a LUMIstar Omega microplate reader luminometer (BMG Labtech, Ortenberg, Germany), and values were normalized to control cells.

### TUNEL assay

Apoptotic DNA fragmentation was evaluated using a commercial TUNEL (Terminal deoxynucleotidyl transferase dUTP nick end labeling) assay kit (Roche Applied Biosciences, Laval, QC, Canada). After miR‑195‑5p transfection (30 or 50 nM), cells were plated in Lab-Tek Chamber Slides 8-wells and grown for 24 h in monitored conditions. Cells were then fixed with 4% paraformaldehyde, permeabilized, and processed following the manufacturer’s guidelines. Fluorescent signals were visualized with a fluorescence microscope (Eclipse Ti2, Nikon Inc., Melville, NY, USA) in randomly selected fields to evaluate the TUNEL‑positive nuclei.

### Annexin V apoptosis assay

HCT116 and HT29 were initially seeded in 6-well plates and harvested 48 h after miR-195-5p mimic transfection. Cells were counted by means of the Countess™ 3 Automated Cell Counter (Thermo Fisher Scientific, Waltham, MA, USA), stained with Muse Annexin V and Dead Cell Reagent (Cytek Biosciences, Fremont, CA, USA) to characterize the distribution of cell populations during the apoptotic process. After 20 min of incubation at room temperature in the dark, apotosis were analyzed using a Guava Muse Cell Analyzer. Data was expressed as percentages of viable, early apoptotic, and late apoptotic cells compared to mock control.

### Immunofluorescence on FFPE tissue sections

For in vivo validation, colon tissues obtained from a previously published AOM/DSS-induced colorectal cancer mouse model treated with miR-195-5p mimic or vehicle control were analyzed. Briefly, colorectal carcinogenesis was induced by azoxymethane (AOM) administration followed by dextran sulfate sodium (DSS) cycles, as previously described by our group [12]. Mice received systemic administration of miR-195-5p mimic, and colonic tissues were collected at sacrifice for molecular analyses. A detailed description of animal procedures, treatment schedule, ethical approvals and experimental design has been reported in our previous study.

Mice formalin‑fixed paraffin‑embedded (FFPE) colon sections (3 µm) were deparaffinized and subjected to antigen retrieval in citrate buffer (pH 6.0) at 98 °C for 30 min. Slides were permeabilized with PBS containing 0.5% Triton X‑100 and blocked with BlockAid Blocking Buffer (Invitrogen, Carlsbad, CA, USA) for 1 h at room temperature. Sections were incubated overnight at 4 °C with primary antibodies targeting XIAP (ab137392, Abcam, Cambridge, UK), BCL2 (sc-7382, Santa Cruz Biotechnology, Inc., Heidelberg, Germany), or Survivin (A2808 Cell Signaling, Technology, Danvers, MA, USA), followed by 1 h incubation with Alexa Fluor‑conjugated secondary antibodies (Thermo Fisher Scientific, Waltham, MA, USA). Nuclei were counterstained using VECTASHIELD Antifade Mounting Medium with DAPI (Vector Laboratories, Newark, CA), and images were acquired using a ZEISS Celldiscoverer 7 imaging system (Zeiss, Jena, Germany). Randomly selected fields were acquired using filters for DAPI, FITC and TRIC using a 10× objective lens. Single channels images were then merged.

### Bioinformatic and statistical analysis

Predicted miR‑195‑5p targets were identified using miRabel [44], miRWalk [45], Tarbase [46], miRGator [47], miRmap [48] and TargetScan [49] tools. Candidate targets were cross-referenced with proteins identified in the apoptosis array and only genes predicted by at least 3 algorithms regulation were selected for further experimental validation.

All experiments were performed in at least three independent replicates. Data are presented as mean ± standard deviation (SD). Statistical analyses were carried out using GraphPad Prism 9 software. Differences between groups were assessed using Student’s t-test. A *p*-value < 0.05 was considered statistically significant.

## Supplementary information

Raw data of Western blot

## Data Availability

Data are within the article and supplementary materials. Raw data of Western blot experiments presented in the study are openly available in FigShare at https://doi.org/10.6084/m9.figshare.32043177.
