# Supplementary material for: miR-195-5p controls apoptotic cascade by regulating the XIAP/BCL2/Survivin network in colorectal cancer
Source: Cell Death Discov. 2026 Jul 28;12:424. doi: 10.1038/s41420-026-03280-y (PMC13694718; doi:10.1038/s41420-026-03280-y)
Supplement: Supplementary file 1 [file 41420_2026_3280_MOESM1_ESM.pdf]

HCT116 Apoptosis array miR-195-5p mimic

Experiment 1 Mock

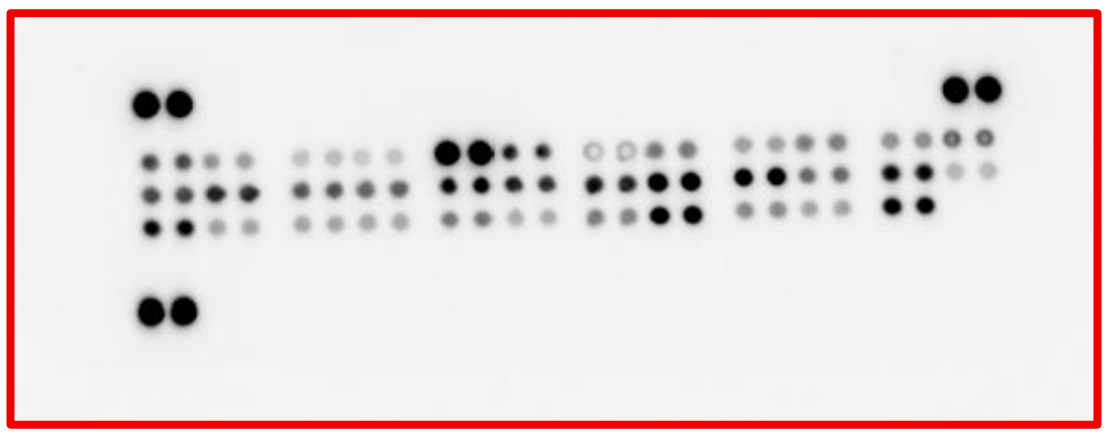

Experiment 2 Mock

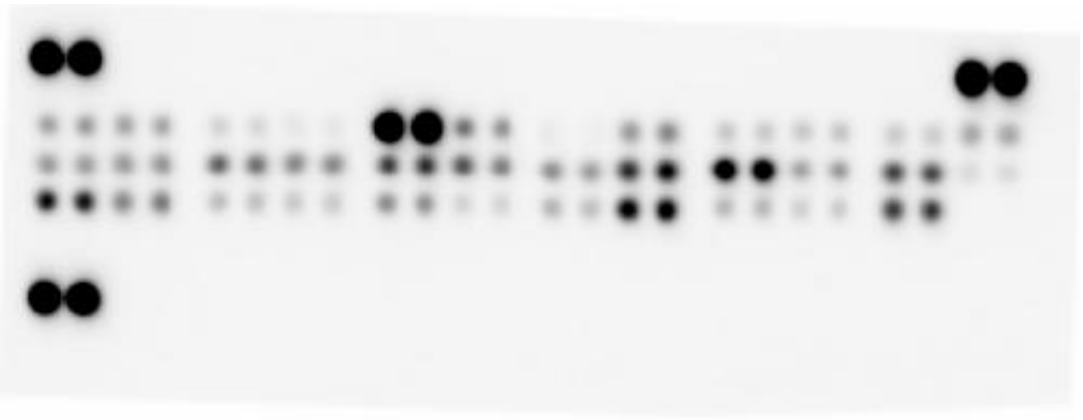

Experiment 3 Mock

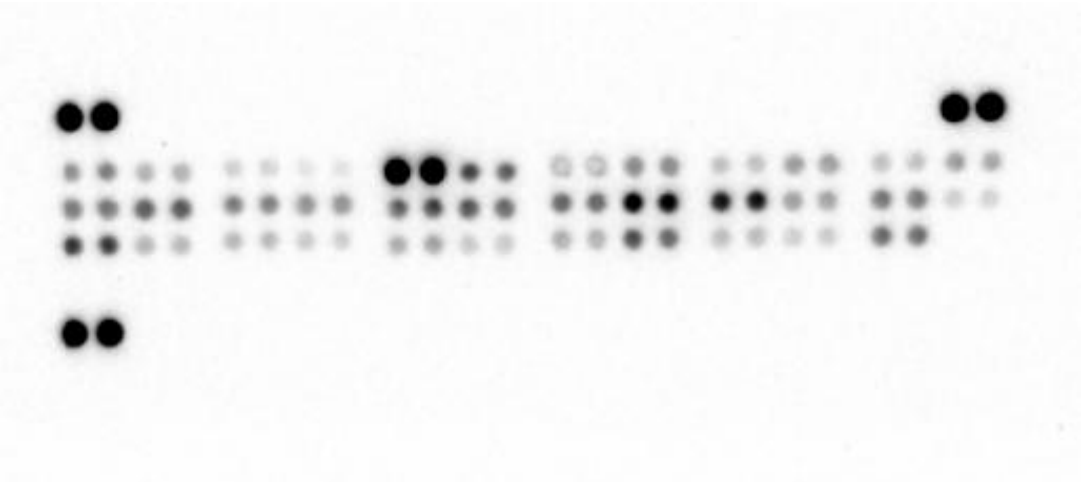

Experiment 1 miR-195-5p 30 nM

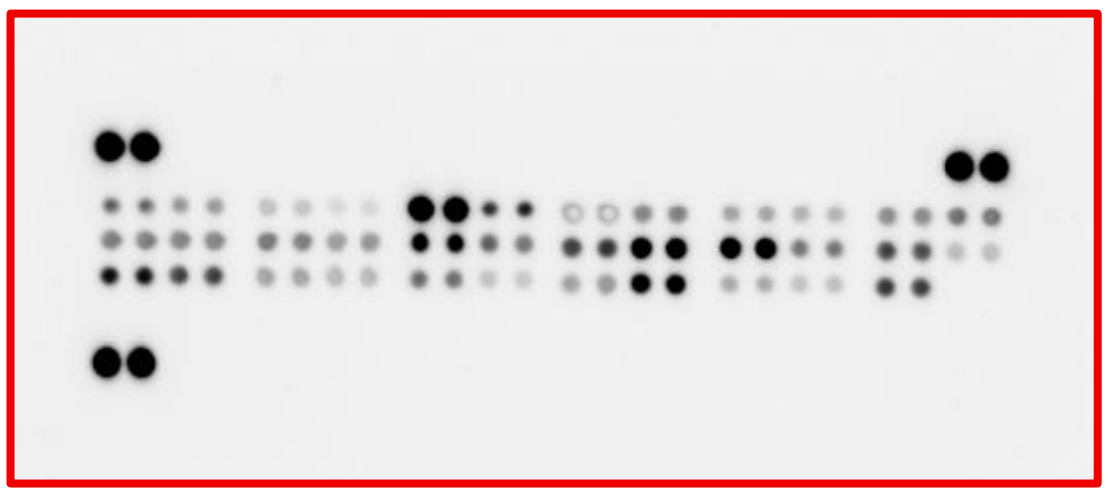

Experiment 2 miR-195-5p 30 nM

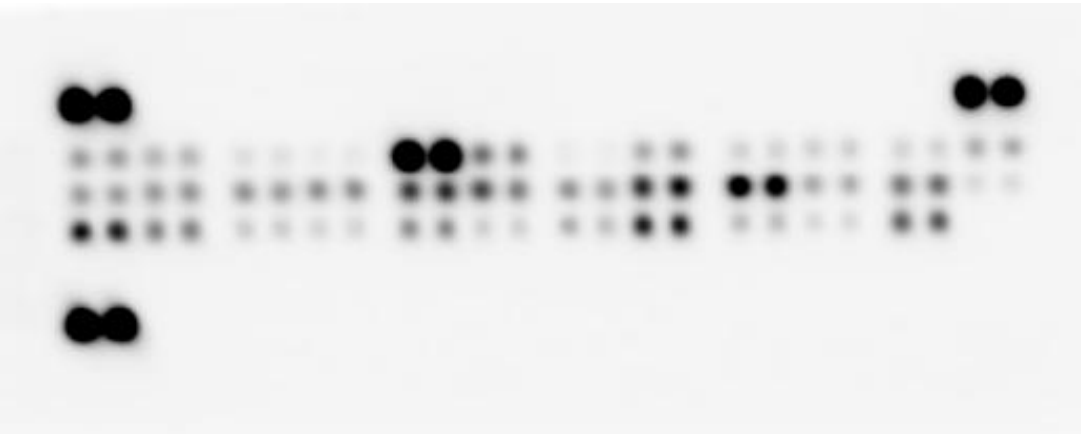

Experiment 3 miR-195-5p 30 nM

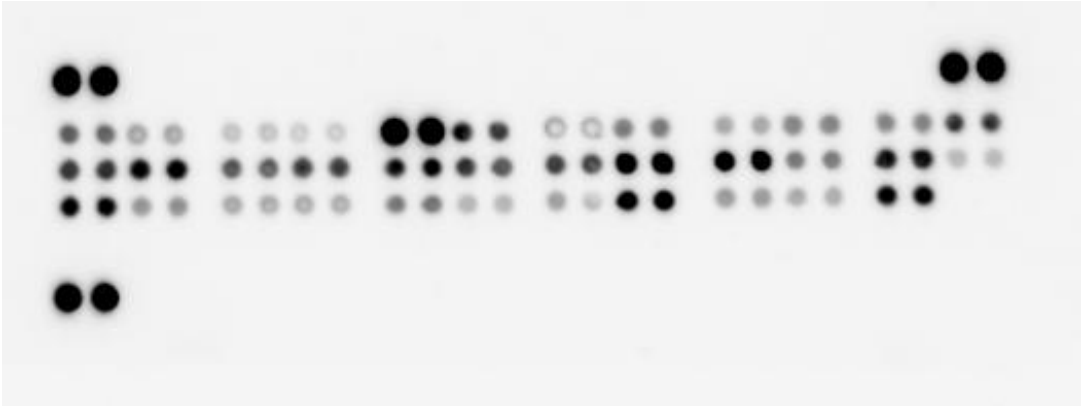

Experiment 1 miR-195-5p 50 nM

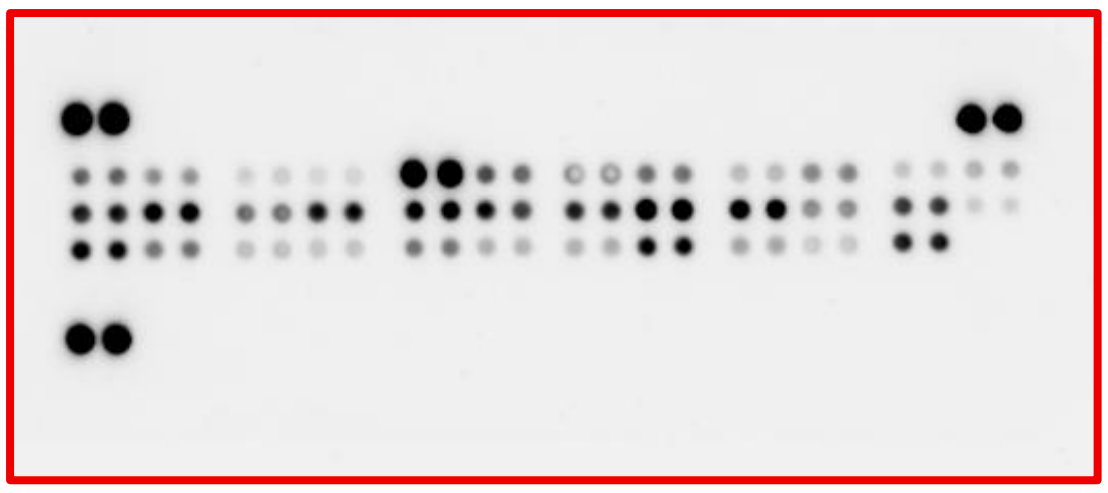

Experiment 2 miR-195-5p 50 nM

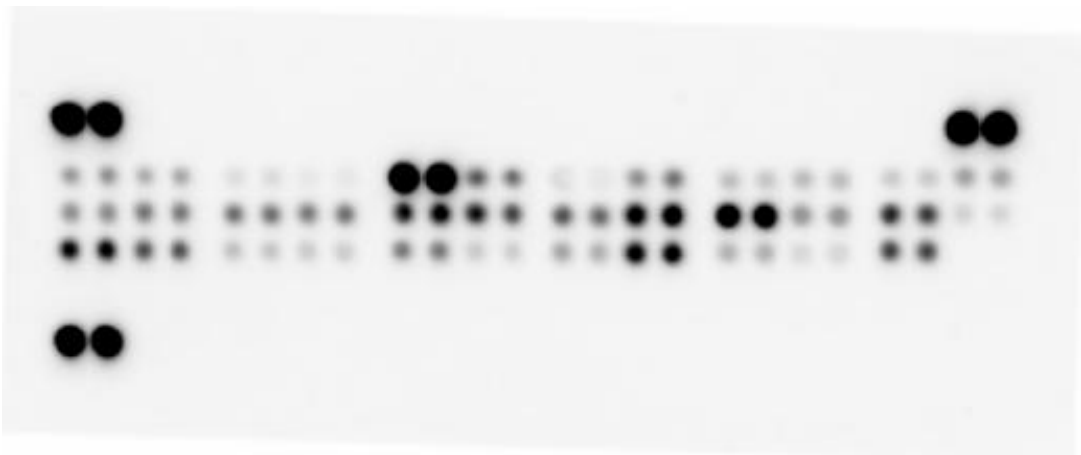

Experiment 3 miR-195-5p 50 nM

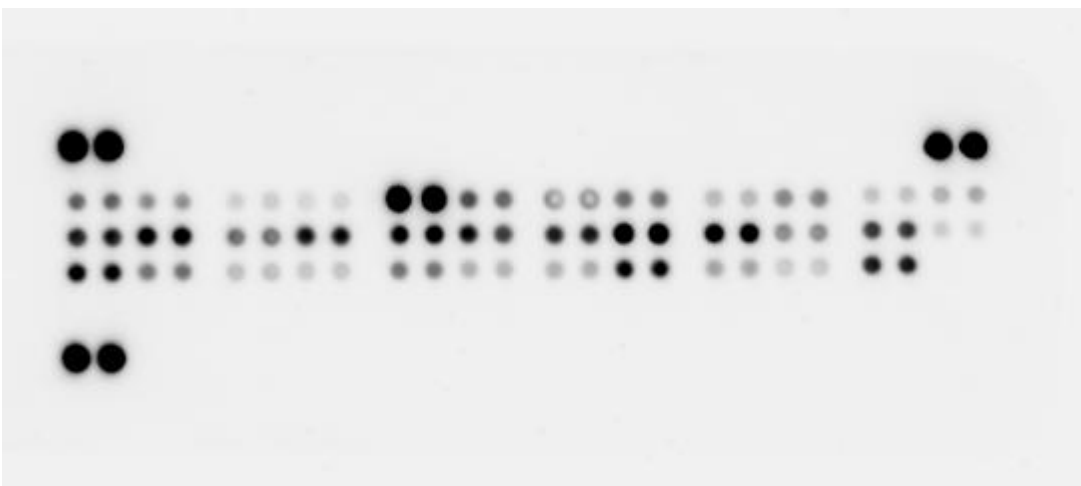

HT29 Apoptosis array miR-195-5p mimic

Experiment 1 Mock

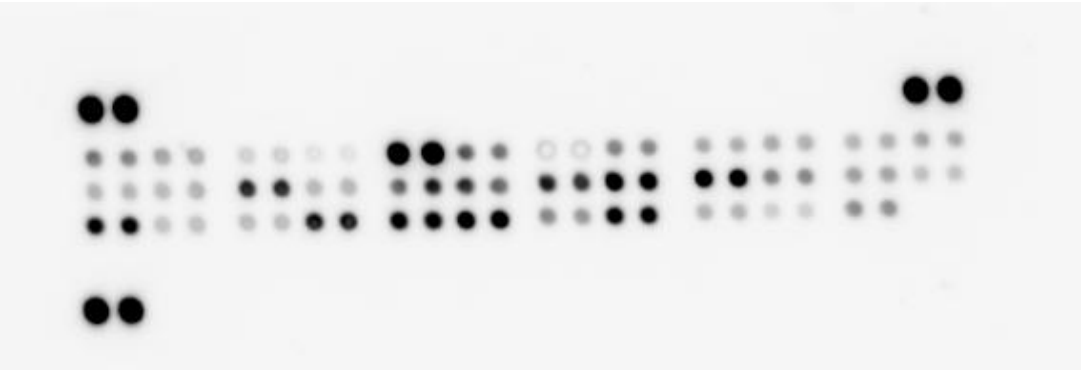

Experiment 2 Mock

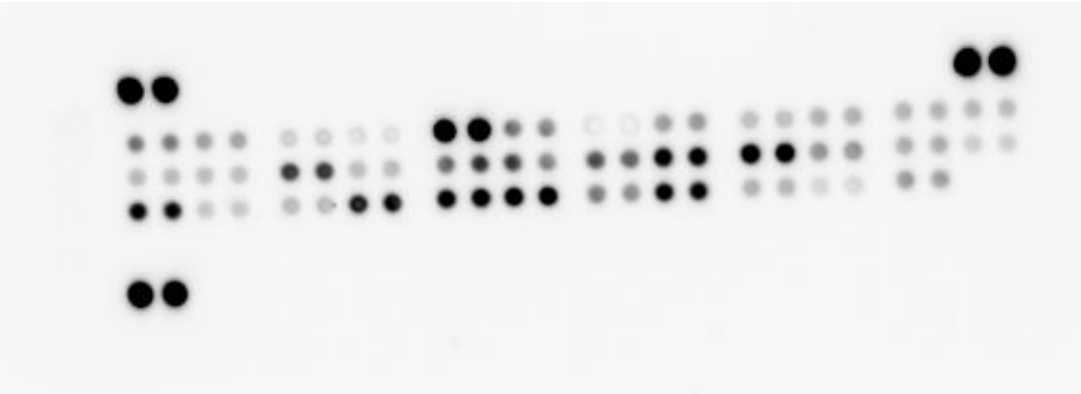

Experiment 3 Mock

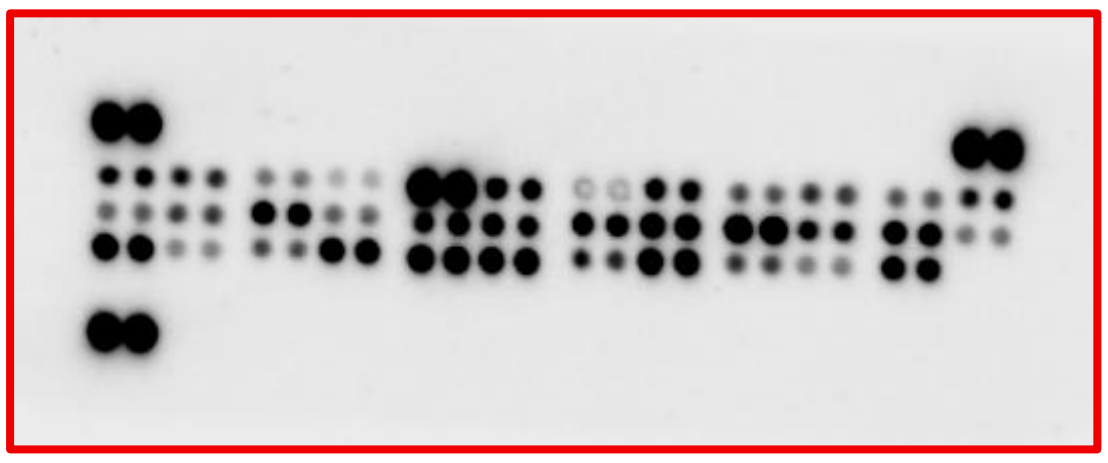

Experiment 1 miR-195-5p 30 nM

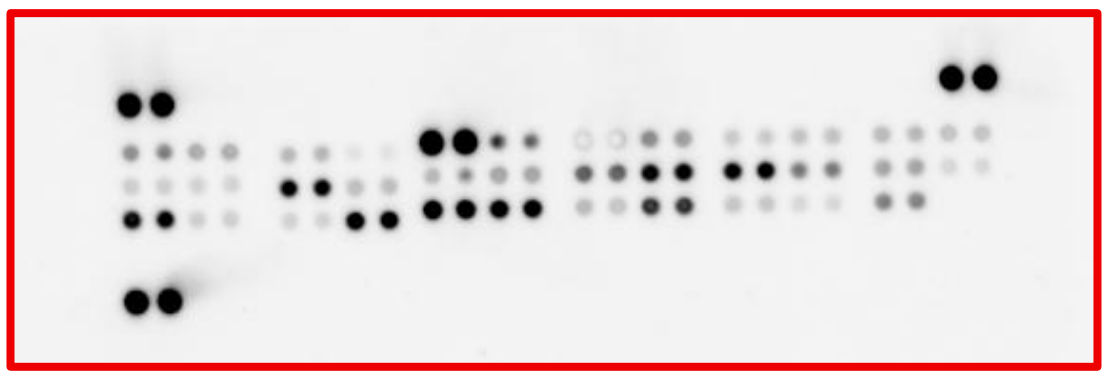

Experiment 2 miR-195-5p 30 nM

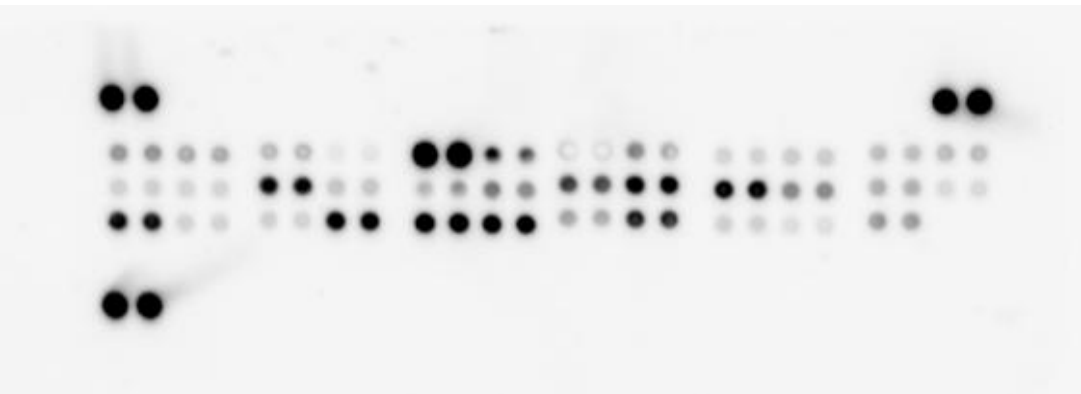

Experiment 3 miR-195-5p 30 nM

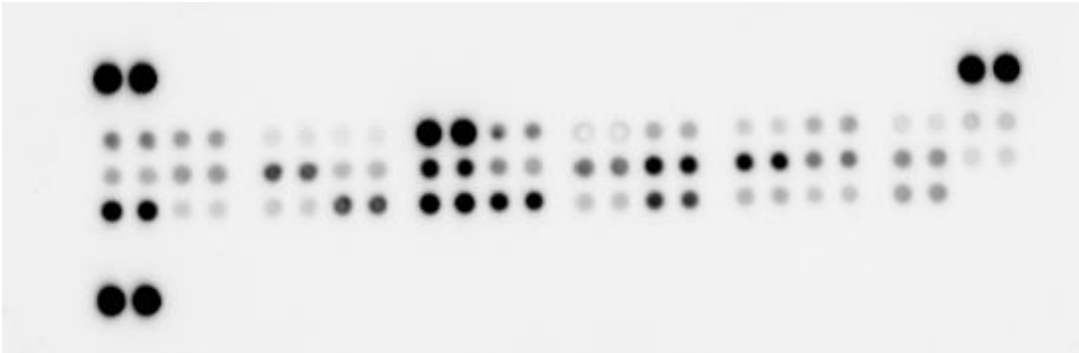

Experiment 1 miR-195-5p 30 nM

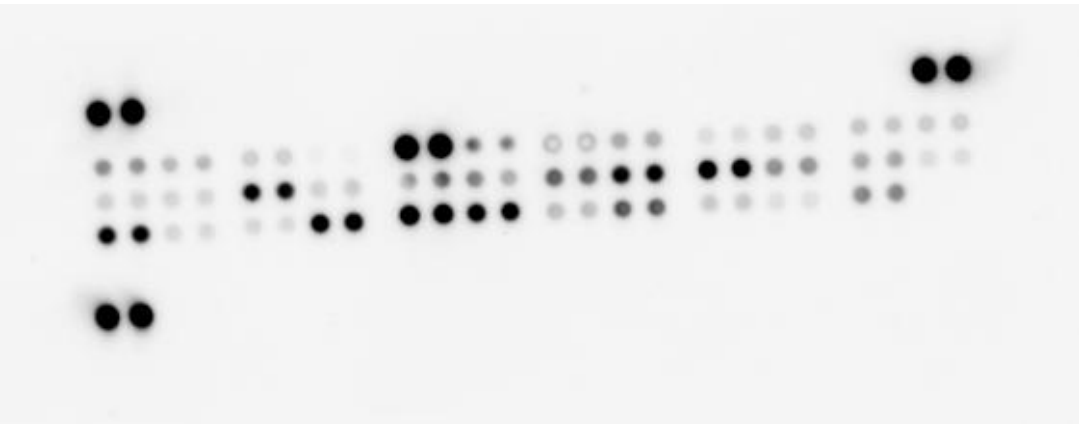

Experiment 2 miR-195-5p 30 nM

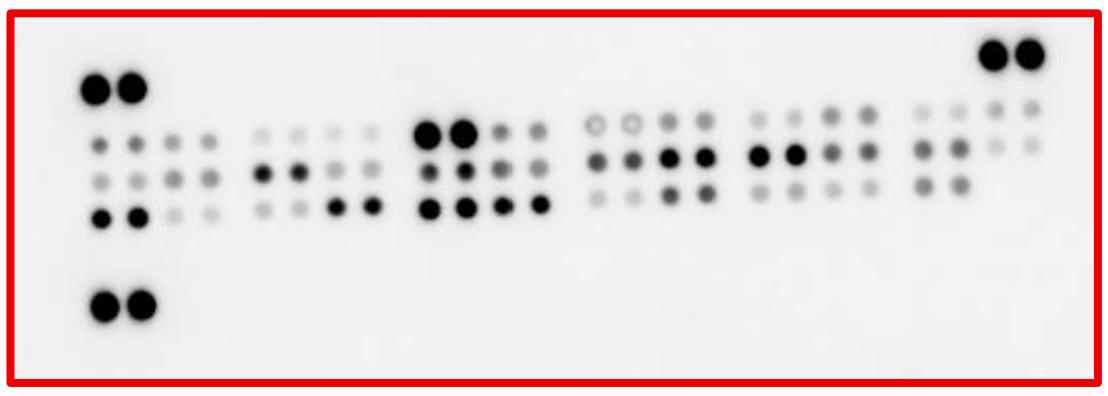

Experiment 3 miR-195-5p 30 nM

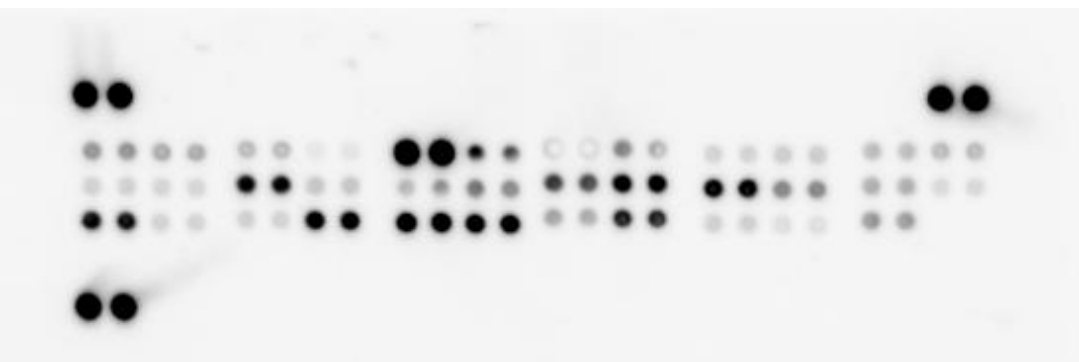

HCT116 Western blot for XIAP

Experiment 1+2 miR-195-5p mimic

XIAP

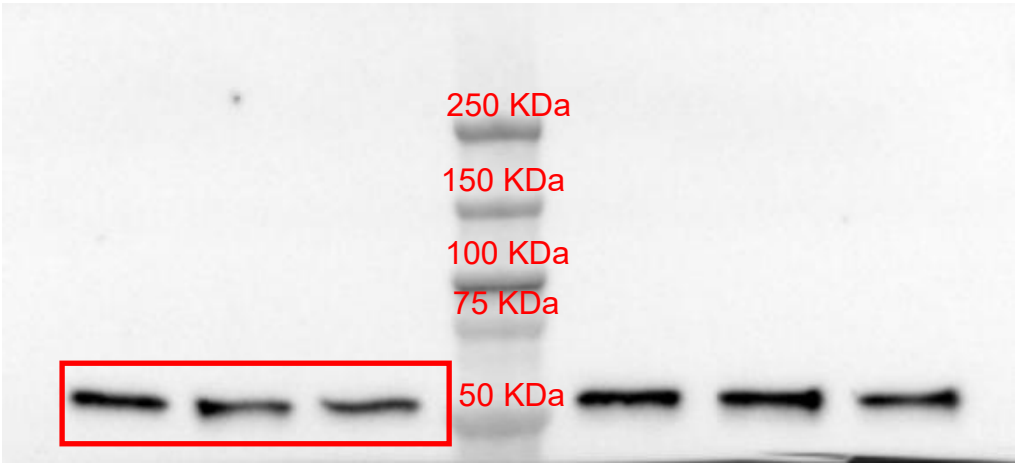

Lane 1 Mock
Lane 2 miR-195-5p mimic 30 nM
Lane 3 miR-195-5p mimic 50 nM
Lane 4 Standard Molecular Weight
Lane 5 Mock
Lane 6 miR-195-5p mimic 30 nM
Lane 7 miR-195-5p mimic 50 nM

ß-tubulin

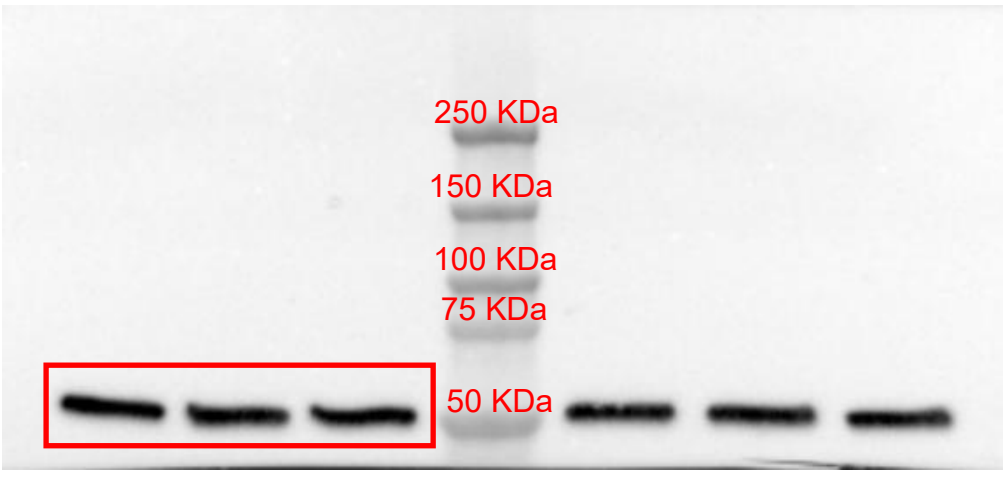

Lane 1 Mock
Lane 2 miR-195-5p mimic 30 nM
Lane 3 miR-195-5p mimic 50 nM
Lane 4 Standard Molecular Weight
Lane 5 Mock
Lane 6 miR-195-5p mimic 30 nM
Lane 7 miR-195-5p mimic 50 nM

Experiment 3 miR-195-5p mimic

XIAP

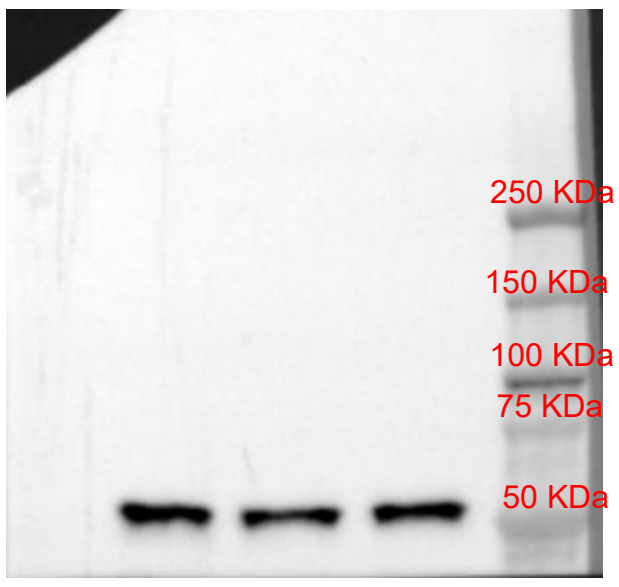

Lane 1 Mock
Lane 2 miR-195-5p mimic 30 nM
Lane 3 miR-195-5p mimic 50 nM
Lane 4 Standard Molecular Weight

ß-tubulin

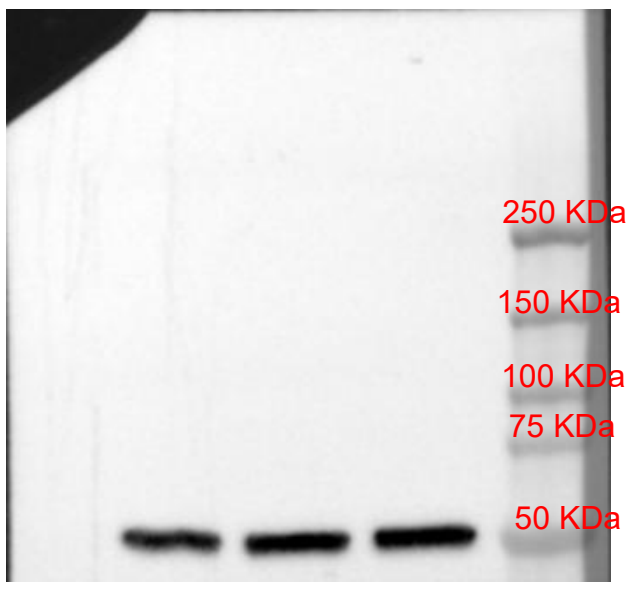

Lane 1 Mock
Lane 2 miR-195-5p mimic 30 nM
Lane 3 miR-195-5p mimic 50 nM
Lane 4 Standard Molecular Weight

Experiment 4 miR-195-5p mimic

XIAP

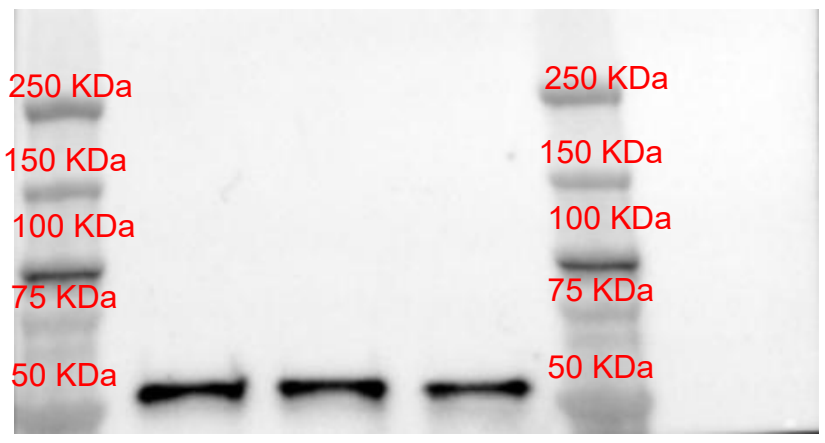

Lane 1 Standard Molecular Weight
Lane 2 Mock
Lane 3 miR-195-5p mimic 30 nM
Lane 4 miR-195-5p mimic 50 nM
Lane 5 Standard Molecular Weight

ß-tubulin

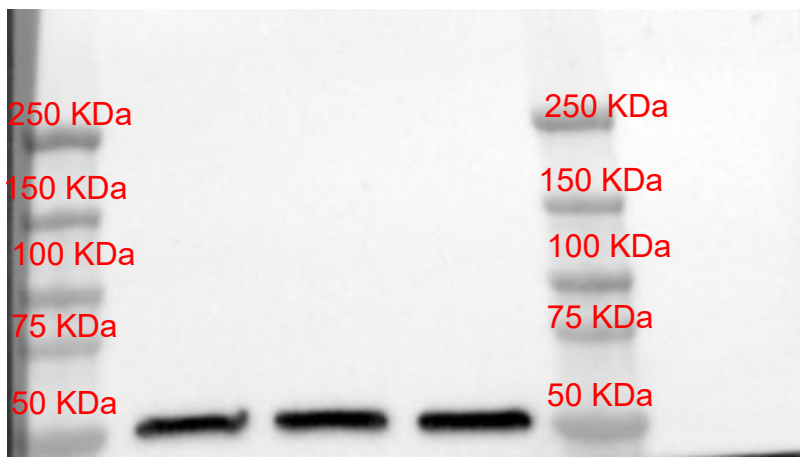

Lane 1 Standard Molecular Weight
Lane 2 Mock
Lane 3 miR-195-5p mimic 30 nM
Lane 4 miR-195-5p mimic 50 nM
Lane 5 Standard Molecular Weight

HCT116 Western blot for BCL2

Experiment 1 miR-195-5p mimic

BCL2

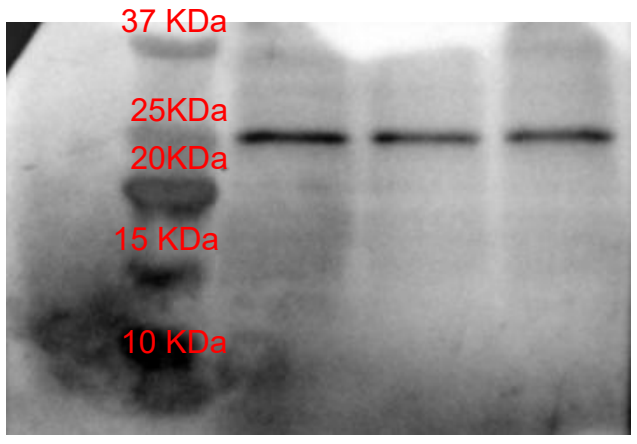

Lane 1 Standard Molecular Weight
Lane 2 Mock
Lane 3 miR-195-5p mimic 30 nM
Lane 4 miR-195-5p mimic 50 nM

ß-tubulin

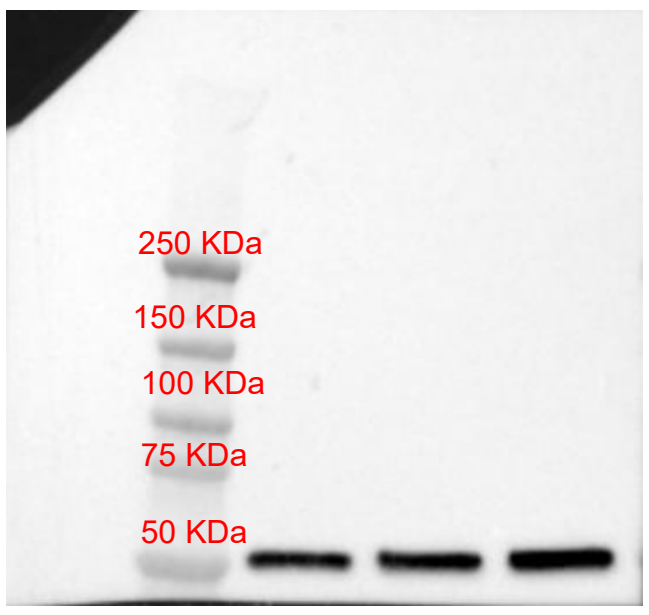

Lane 1 Standard Molecular Weight
Lane 2 Mock
Lane 3 miR-195-5p mimic 30 nM
Lane 4 miR-195-5p mimic 50 nM

Experiment 2 miR-195-5p mimic

BCL2

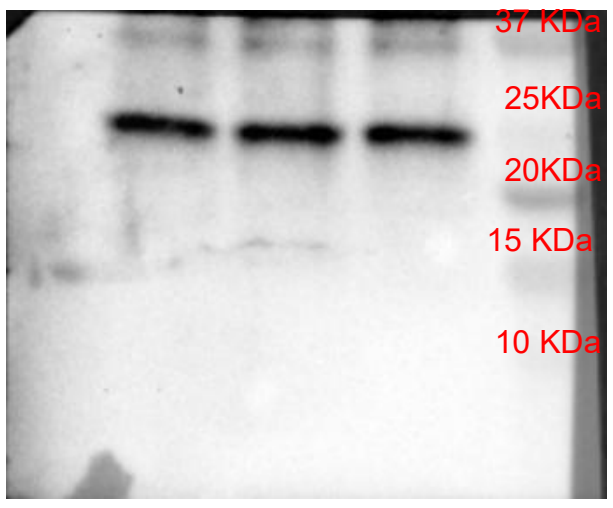

Lane 1 Mock
Lane 2 miR-195-5p mimic 30 nM
Lane 3 miR-195-5p mimic 50 nM
Lane 4 Standard Molecular Weight

ß-tubulin

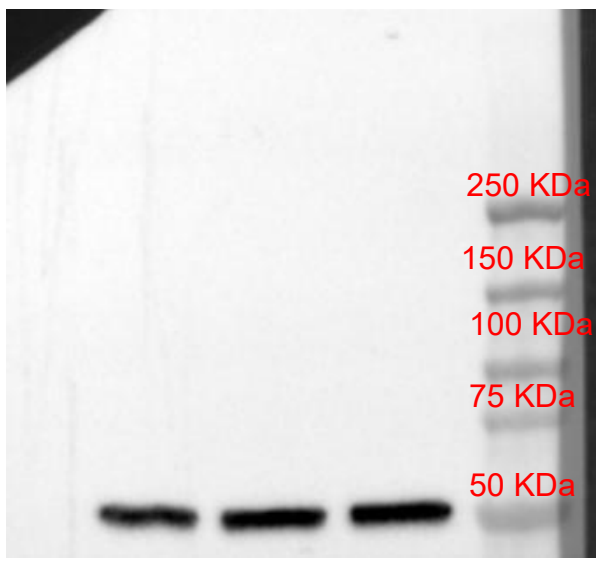

Lane 1 Mock
Lane 2 miR-195-5p mimic 30 nM
Lane 3 miR-195-5p mimic 50 nM
Lane 4 Standard Molecular Weight

Experiment 3 miR-195-5p mimic

BCL2

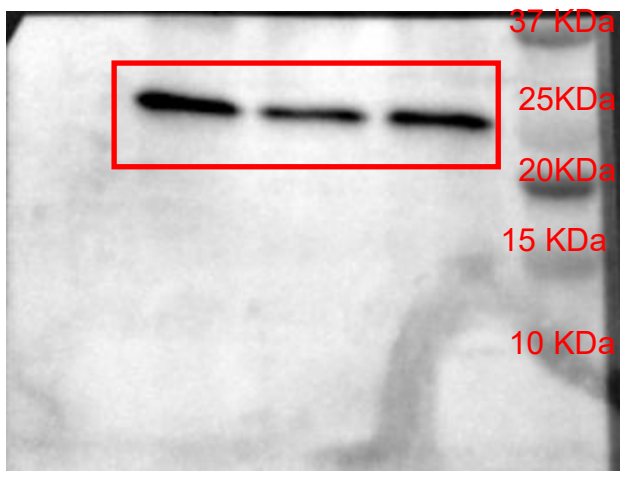

Lane 1 Mock
Lane 2 miR-195-5p mimic 30 nM
Lane 3 miR-195-5p mimic 50 nM
Lane 4 Standard Molecular Weight

ß-tubulin

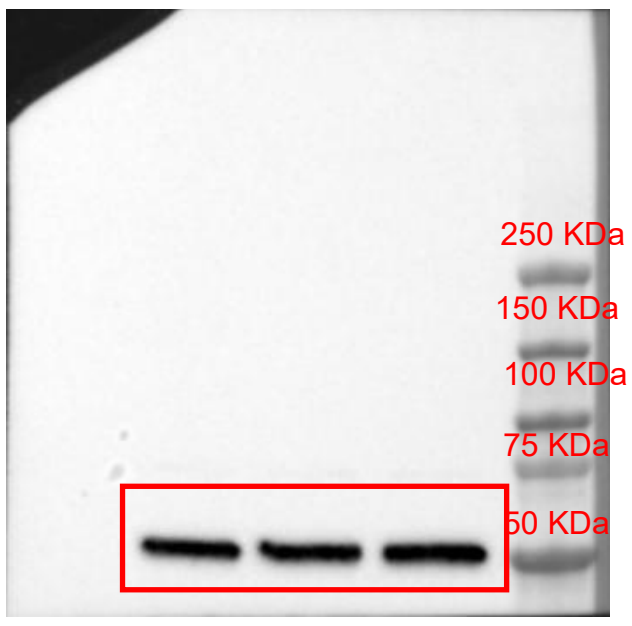

Lane 1 Mock
Lane 2 miR-195-5p mimic 30 nM
Lane 3 miR-195-5p mimic 50 nM
Lane 4 Standard Molecular Weight

Experiment 4 miR-195-5p mimic

BCL2

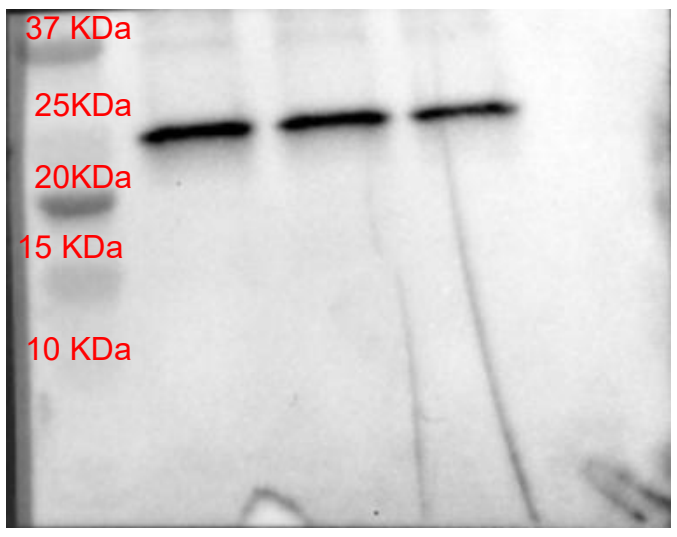

Lane 1 Standard Molecular Weight
Lane 2 Mock
Lane 3 miR-195-5p mimic 30 nM
Lane 4 miR-195-5p mimic 50 nM

ß-tubulin

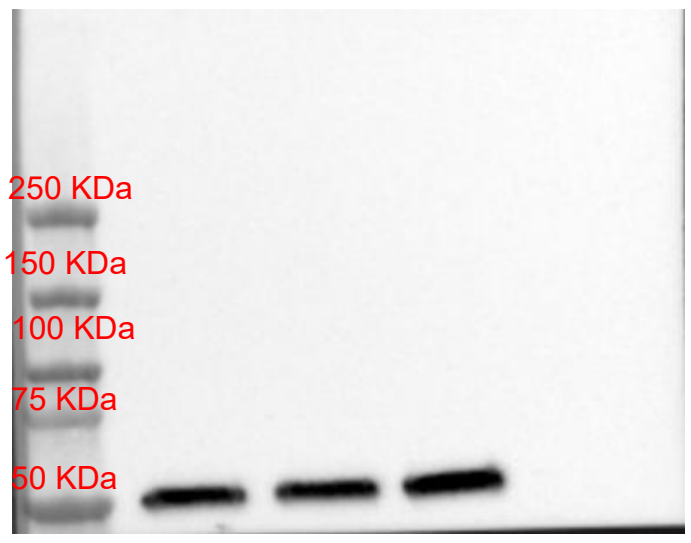

Lane 1 Standard Molecular Weight
Lane 2 Mock
Lane 3 miR-195-5p mimic 30 nM
Lane 4 miR-195-5p mimic 50 nM

HCT116 Western blot for Survivin

Experiment 1 miR-195-5p mimic

Survivin

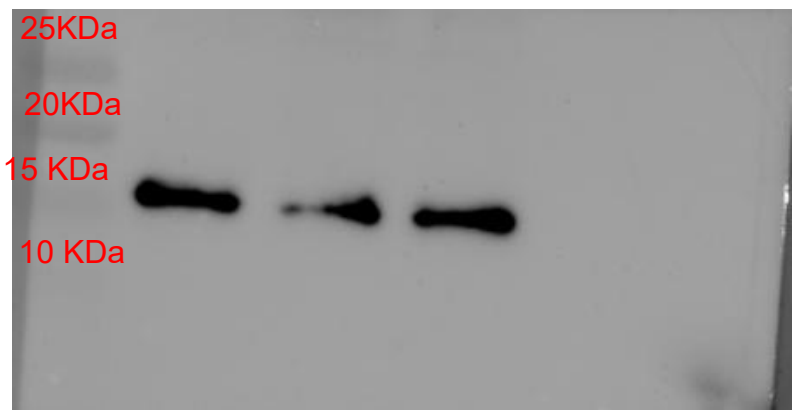

Lane 1 Standard Molecular Weight
Lane 2 Mock
Lane 3 miR-195-5p mimic 30 nM
Lane 4 miR-195-5p mimic 50 nM

ß-tubulin

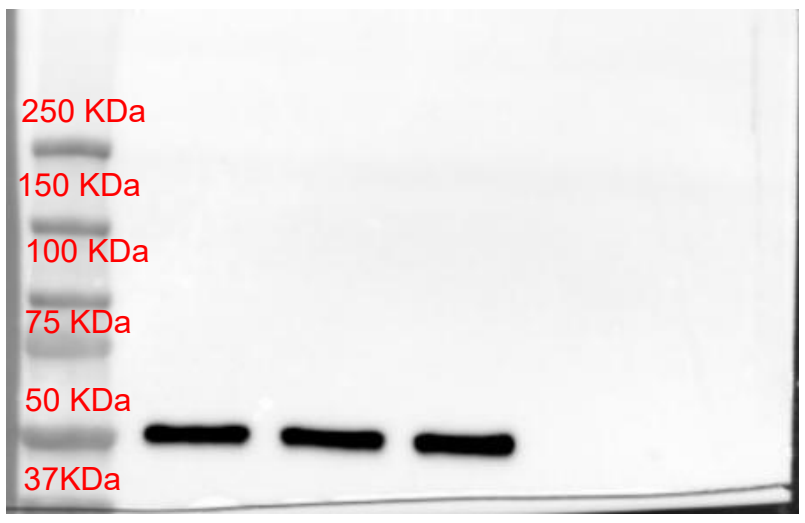

Lane 1 Standard Molecular Weight
Lane 2 Mock
Lane 3 miR-195-5p mimic 30 nM
Lane 4 miR-195-5p mimic 50 nM

Experiment 2 miR-195-5p mimic

Survivin

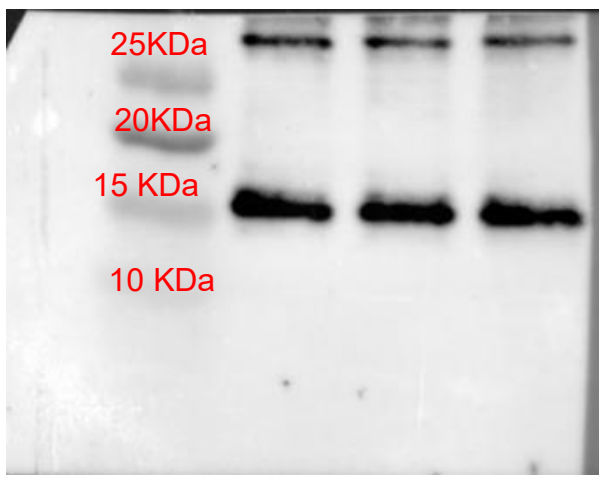

Lane 1 Standard Molecular Weight
Lane 2 Mock
Lane 3 miR-195-5p mimic 30 nM
Lane 4 miR-195-5p mimic 50 nM

ß-tubulin

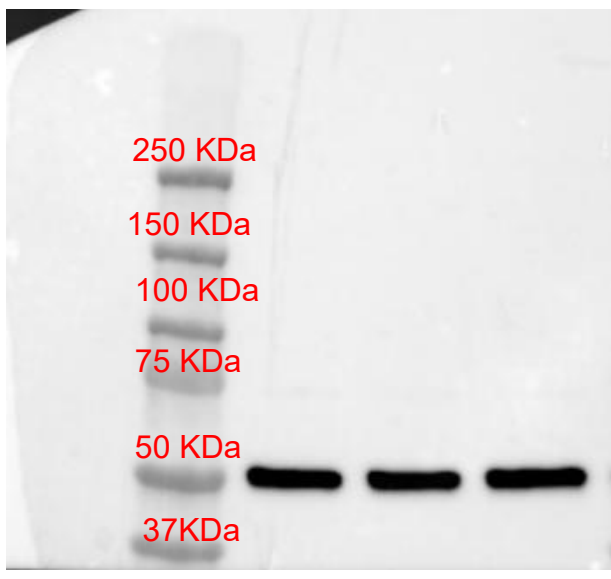

Lane 1 Standard Molecular Weight
Lane 2 Mock
Lane 3 miR-195-5p mimic 30 nM
Lane 4 miR-195-5p mimic 50 nM

Experiment 3 miR-195-5p mimic

Survivin

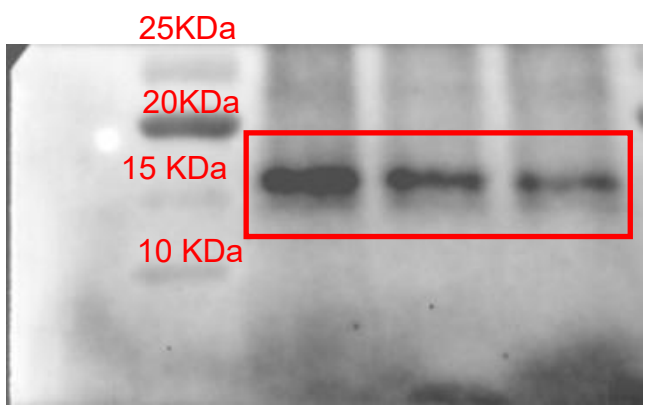

Lane 1 Standard Molecular Weight
Lane 2 Mock
Lane 3 miR-195-5p mimic 30 nM
Lane 4 miR-195-5p mimic 50 nM

ß-tubulin

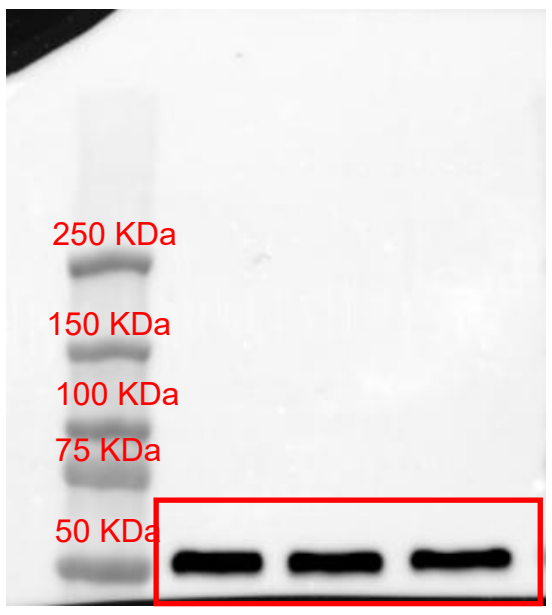

Lane 1 Standard Molecular Weight
Lane 2 Mock
Lane 3 miR-195-5p mimic 30 nM
Lane 4 miR-195-5p mimic 50 nM

Experiment 4 miR-195-5p mimic

Survivin

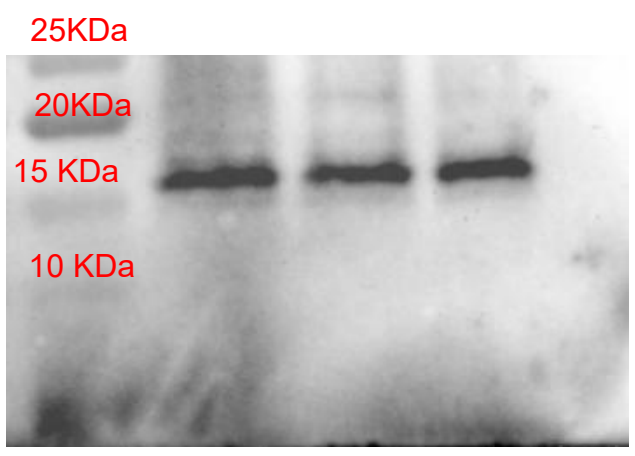

Lane 1 Standard Molecular Weight
Lane 2 Mock
Lane 3 miR-195-5p mimic 30 nM
Lane 4 miR-195-5p mimic 50 nM

ß-tubulin

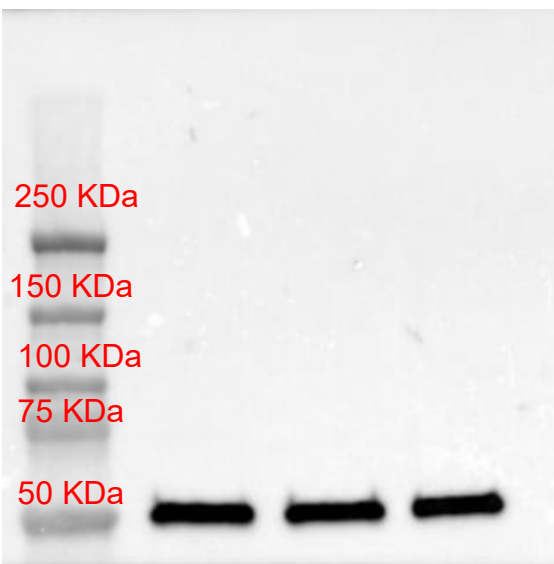

Lane 1 Standard Molecular Weight
Lane 2 Mock
Lane 3 miR-195-5p mimic 30 nM
Lane 4 miR-195-5p mimic 50 nM

HT29 Western blot for XIAP

Experiment 1 miR-195-5p mimic

XIAP

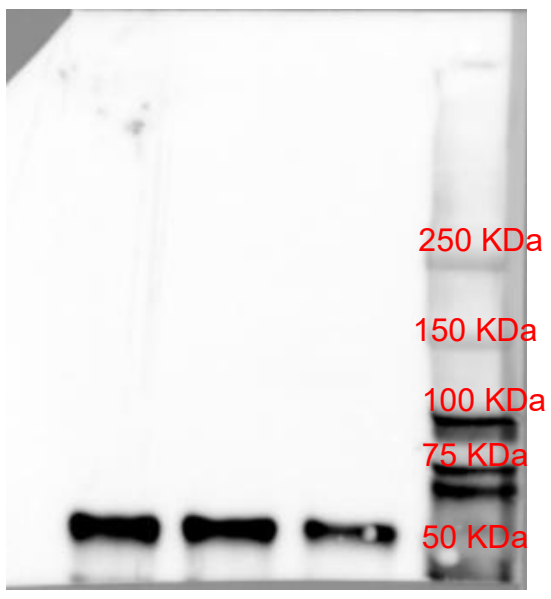

Lane 1 Mock
Lane 2 miR-195-5p mimic 30 nM
Lane 3 miR-195-5p mimic 50 nM
Lane 4 Standard Molecular Weight

ß-tubulin

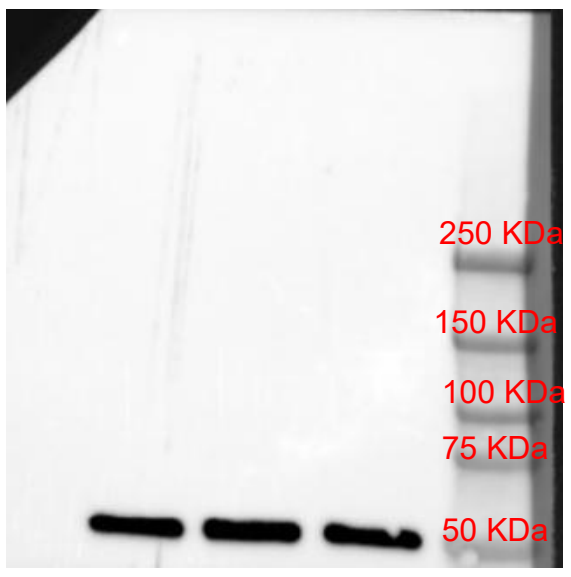

Lane 1 Mock
Lane 2 miR-195-5p mimic 30 nM
Lane 3 miR-195-5p mimic 50 nM
Lane 4 Standard Molecular Weight

Experiment 2 miR-195-5p mimic

XIAP

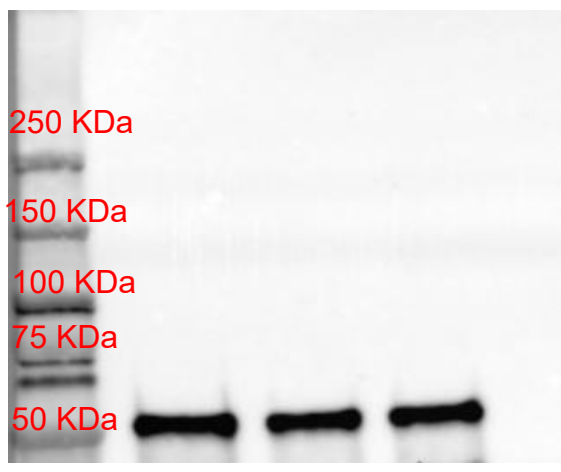

Lane 1 Standard Molecular Weight
Lane 2 Mock
Lane 3 miR-195-5p mimic 30 nM
Lane 4 miR-195-5p mimic 50 nM

ß-tubulin

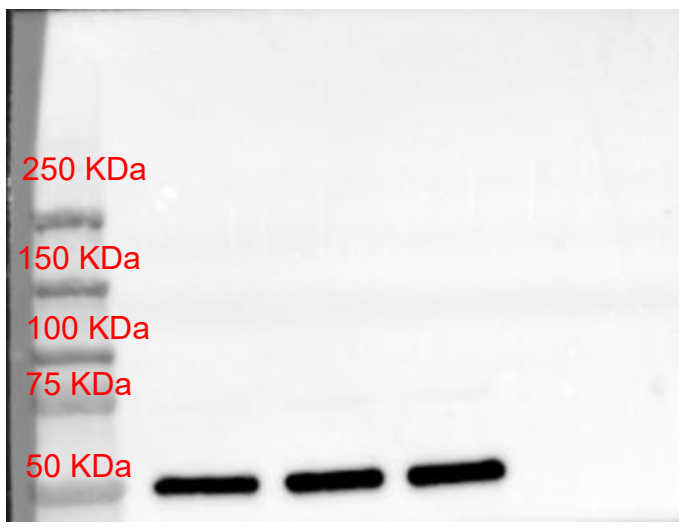

Lane 1 Standard Molecular Weight
Lane 2 Mock
Lane 3 miR-195-5p mimic 30 nM
Lane 4 miR-195-5p mimic 50 nM

Experiment 3 miR-195-5p mimic

XIAP

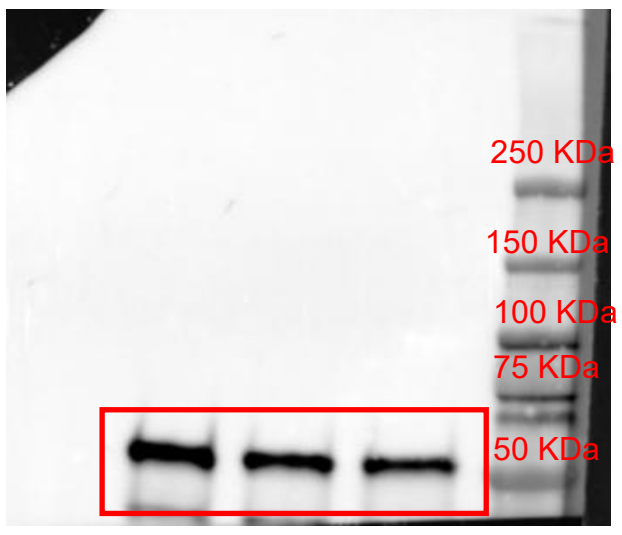

Lane 1 Mock
Lane 2 miR-195-5p mimic 30 nM
Lane 3 miR-195-5p mimic 50 nM
Lane 4 Standard Molecular Weight

ß-tubulin

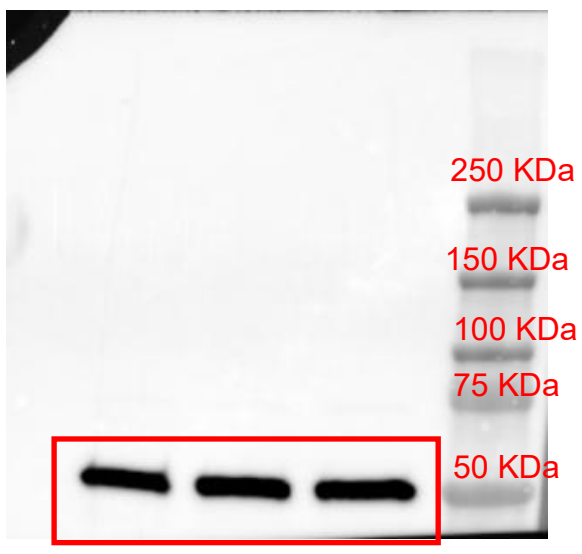

Lane 1 Mock
Lane 2 miR-195-5p mimic 30 nM
Lane 3 miR-195-5p mimic 50 nM
Lane 4 Standard Molecular Weight

HT29 Western blot for BCL2

Experiment 1 miR-195-5p mimic

BCL2

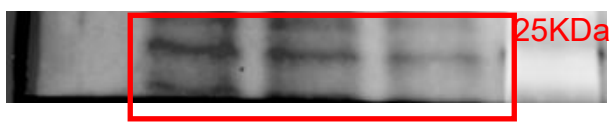

Lane 1 Mock
Lane 2 miR-195-5p mimic 30 nM
Lane 3 miR-195-5p mimic 50 nM
Lane 4 Standard Molecular Weight

ß-tubulin

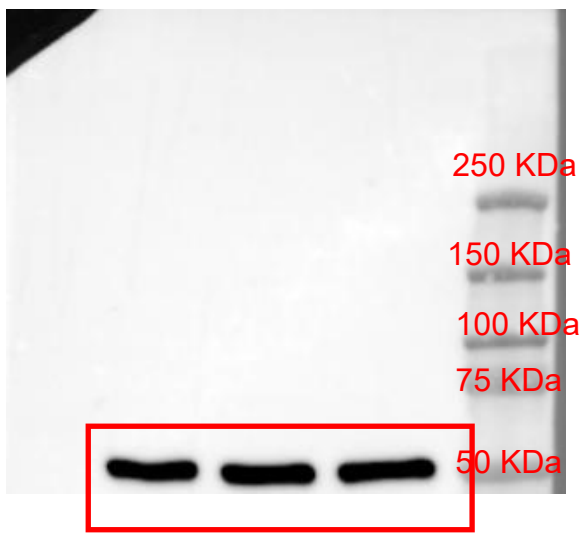

Lane 1 Mock
Lane 2 miR-195-5p mimic 30 nM
Lane 3 miR-195-5p mimic 50 nM
Lane 4 Standard Molecular Weight

Experiment 2 miR-195-5p mimic

BCL2

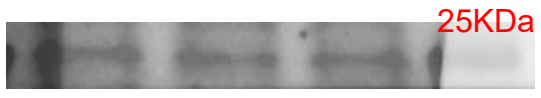

Lane 1 Mock
Lane 2 miR-195-5p mimic 30 nM
Lane 3 miR-195-5p mimic 50 nM
Lane 4 Standard Molecular Weight

ß-tubulin

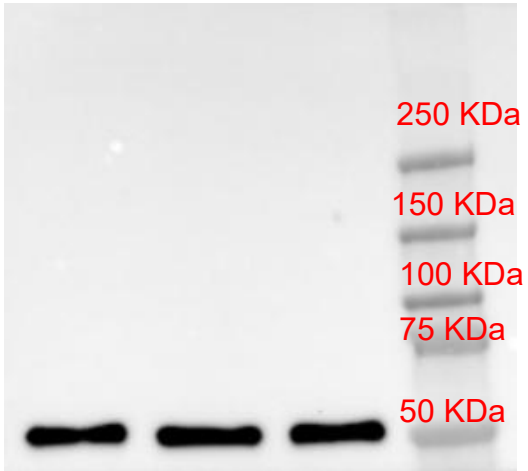

Lane 1 Mock
Lane 2 miR-195-5p mimic 30 nM
Lane 3 miR-195-5p mimic 50 nM
Lane 4 Standard Molecular Weight

Experiment 3 miR-195-5p mimic

BCL2

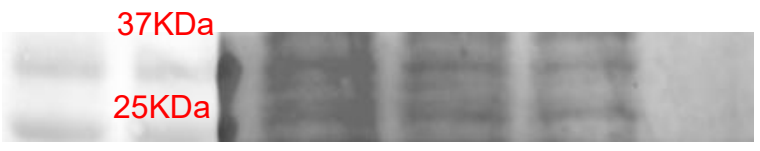

Lane 1 Standard Molecular Weight
Lane 2 Mock
Lane 3 miR-195-5p mimic 30 nM
Lane 4 miR-195-5p mimic 50 nM

ß-tubulin

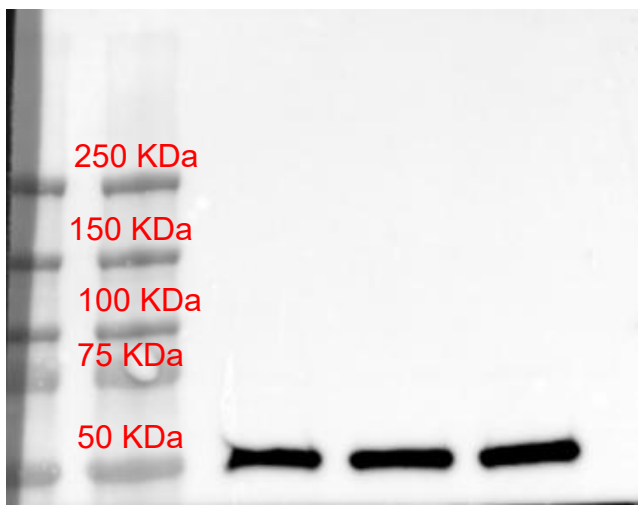

Lane 1 Standard Molecular Weight
Lane 2 Mock
Lane 3 miR-195-5p mimic 30 nM
Lane 4 miR-195-5p mimic 50 nM

HT29 Western blot for Survivin

Experiment 1 miR-195-5p mimic

Survivin

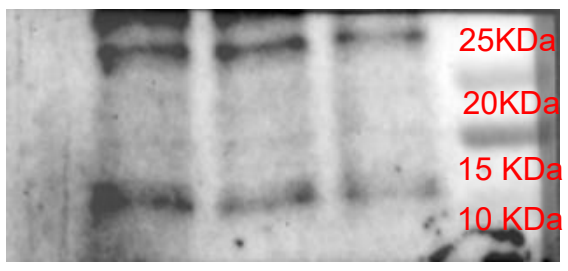

Lane 1 Mock
Lane 2 miR-195-5p mimic 30 nM
Lane 3 miR-195-5p mimic 50 nM
Lane 4 Standard Molecular Weight

ß-tubulin

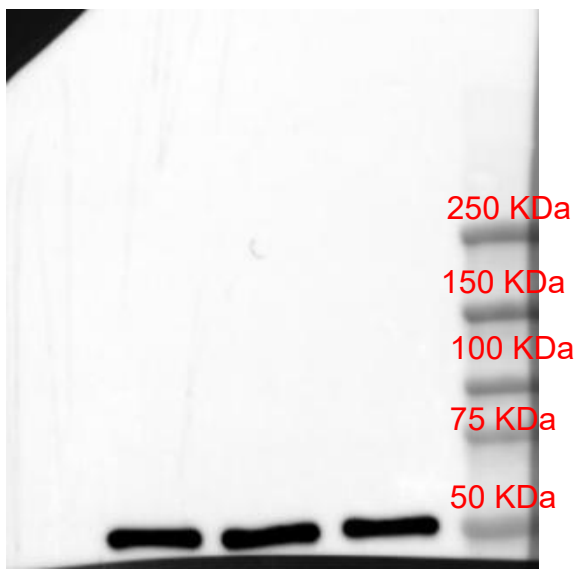

Lane 1 Mock
Lane 2 miR-195-5p mimic 30 nM
Lane 3 miR-195-5p mimic 50 nM
Lane 4 Standard Molecular Weight

Experiment 2 miR-195-5p mimic

Survivin

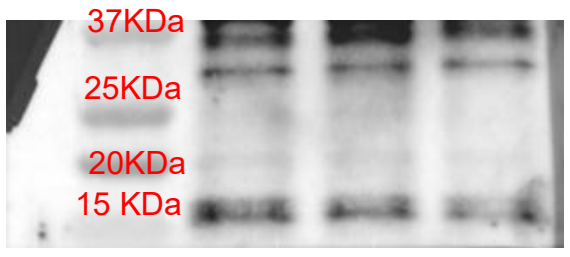

Lane 1 Standard Molecular Weight
Lane 2 Mock
Lane 3 miR-195-5p mimic 30 nM
Lane 4 miR-195-5p mimic 50 nM

ß-tubulin

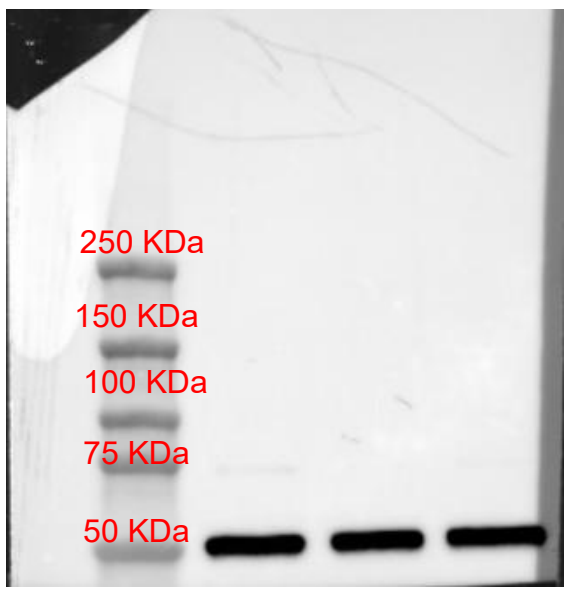

Lane 1 Standard Molecular Weight
Lane 2 Mock
Lane 3 miR-195-5p mimic 30 nM
Lane 4 miR-195-5p mimic 50 nM

Experiment 3 miR-195-5p mimic

Survivin

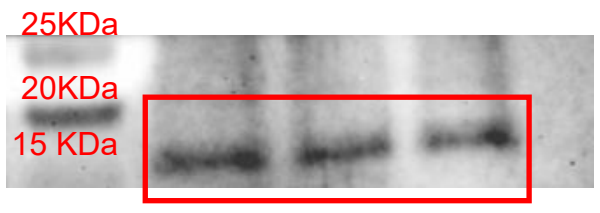

Lane 1 Standard Molecular Weight
Lane 2 Mock
Lane 3 miR-195-5p mimic 30 nM
Lane 4 miR-195-5p mimic 50 nM

ß-tubulin

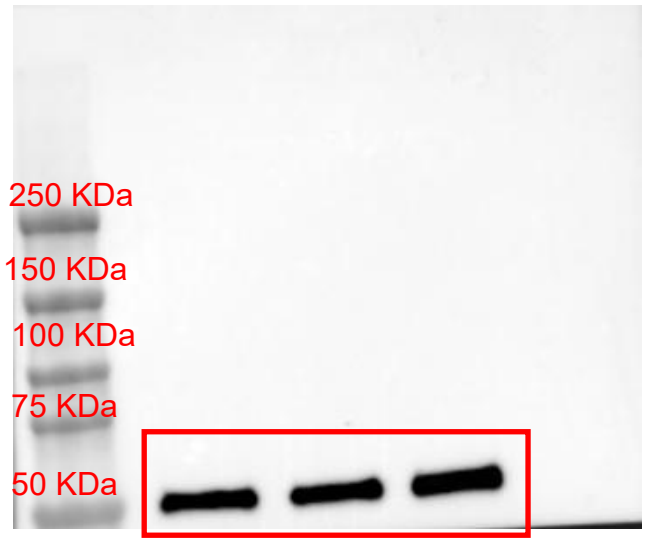

Lane 1 Standard Molecular Weight
Lane 2 Mock
Lane 3 miR-195-5p mimic 30 nM
Lane 4 miR-195-5p mimic 50 nM
